# Distinct chikungunya virus polymerase palm subdomains contribute to viral protein accumulation and virion production

**DOI:** 10.1371/journal.ppat.1011972

**Published:** 2024-10-14

**Authors:** Marie-France Martin, Boris Bonaventure, Nia E. McCray, Olve B. Peersen, Kathryn Rozen-Gagnon, Kenneth A. Stapleford

**Affiliations:** 1 Department of Microbiology, New York University Grossman School of Medicine, New York, New York, United States of America; 2 Department of Microbiology, Icahn School of Medicine at Mount Sinai, New York, New York, United States of America; 3 Department of Biochemistry & Molecular Biology, Colorado State University, Fort Collins, Colorado, United States of America; 4 Department of Molecular Genetics, University of Toronto, Toronto, Canada; University of North Carolina at Chapel Hill, UNITED STATES OF AMERICA

## Abstract

Alphaviruses encode an error-prone RNA-dependent RNA polymerase (RdRp), nsP4, required for genome synthesis, yet how the RdRp functions in the complete alphavirus life cycle is not well-defined. Previous work using chikungunya virus has established the importance of the nsP4 residue cysteine 483 in replication. Given the location of residue C483 in the nsP4 palm domain, we hypothesized that other residues within this domain and surrounding subdomains would also contribute to polymerase function. To test this hypothesis, we designed a panel of nsP4 variants via homology modeling based on the coxsackievirus B3 3D polymerase. We rescued each variant in mammalian and mosquito cells and discovered that the palm domain and ring finger subdomain contribute to host-specific replication. In C6/36 cells, we found that while the nsP4 variants had replicase function similar to that of wild-type CHIKV, many variants presented changes in protein accumulation and virion production even when viral nonstructural and structural proteins were produced. Finally, we found that WT CHIKV and nsP4 variant replication and protein production could be enhanced in mammalian cells at 28°C, yet growing virus under these conditions led to changes in virus infectivity. Taken together, these studies highlight that distinct nsP4 subdomains are required for proper RNA transcription and translation, having major effects on virion production.

## Author summary

Chikungunya virus (CHIKV) is a re-emerging alphavirus transmitted to humans by mosquitoes. Its replication relies on a polymerase that incorporates a significant number of errors in the new genomes, making it a good candidate to develop vaccines or antiviral strategies. However, little is known on alphavirus polymerase function in alternate hosts. To begin to understand how the CHIKV polymerase nsP4 functions, we designed a panel of nsP4 variants taking advantage of the conservation of polymerase structure across positive-strand RNA viruses. We discovered that the palm domain and ring finger subdomain of the polymerase were involved in host-specific RNA transcription and virion production. Taken together, these findings add further evidence to the crucial impact of the core palm domain of CHIKV polymerase not only on the transcription of the RNA itself, but also on the complete viral life cycle.

## Introduction

Chikungunya virus (CHIKV) is a re-emerging mosquito-borne virus, belonging to the *Togaviridae* family and the *Alphavirus* genus. Alphaviruses, which include both arthritogenic viruses like CHIKV and Mayaro virus (MAYV), and encephalitic viruses like eastern equine encephalitis virus (EEEV) and Venezuelan equine encephalitis virus (VEEV), can cause devastating disease and explosive outbreaks. Despite the severity of these diseases, there are limited vaccines or therapeutic strategies targeting alphaviruses. This fact, coupled with global climate change driving mosquito spread and the significant economic and social burden of alphaviral disease, highlights the need to better study how alphaviruses replicate at the molecular level [1–4].

CHIKV is an enveloped virus that possesses a ~12 kb positive-sense single-stranded RNA genome flanked by a capped 5’-untranslated region (UTR) and a polyadenylated 3’-UTR. The genome contains two open-reading frames (ORF). The first ORF encodes for the nonstructural polyprotein P1234, that gives rise upon cleavage to the nonstructural proteins nsP1, nsP2, nsP3, and nsP4. The second ORF is under the control of an internal subgenomic promoter and encodes for the structural proteins capsid, E3, E2, 6K/TF and E1 [5]. nsP1 is the methyl/guanylyltransferase responsible for capping new viral genomes [6], membrane curvature during replication complex formation [7] and anchoring of the replication complexes, also called spherules, at the plasma membrane [8,9]. nsP2 has protease, helicase and NTPase activities [10–12] and controls fidelity in synergy with nsP4 [13]. nsP3 possesses both a N-terminal ADP-ribose binding and hydrolase activities [14], as well as a hyper-variable C-terminal domain involved in binding cellular co-factors [15]. Finally, nsP4 is the RNA-dependent RNA polymerase (RdRp) and possesses an acetyltransferase activity [16], which is required to generate the polyadenylated tail at the 3’ end of the new viral genomes.

In addition to RNA polymerization, CHIKV nsP4 is a key contributor to the intrinsic viral replication fidelity as previously characterized with the cysteine at position 483 [17,18]. Fidelity is defined by the ability of a polymerase to incorporate, based on a template, the correct nucleotide over a non-correct nucleotide during replication. Mutations at the position 483 of nsP4 result in varying phenotypes depending on the nature of the introduced residue. Whereas C483Y confers a high-fidelity phenotype to the polymerase [17], substitution with a glycine, C483G, results in a low-fidelity polymerase variant [18]. Interestingly, both high- and low-fidelity polymerase variants are attenuated in mosquitoes and mice [17,18], highlighting the fine tuning of polymerase fidelity.

Recent work has unraveled the structures of alphavirus nsP4 and the replication complex [19,20]. The elegant study by Tan *et al*, demonstrated that CHIKV active replicase is composed of a dodecameric ring of nsP1 sitting at the neck of each spherule, at the center of which, one nsP2 molecule takes place on the cytoplasmic side, itself faced by one nsP4 molecule on the spherule side [19]. Whereas these studies provide important structural insight on the CHIKV polymerase, nsP4 function is still not well-characterized. We suspect that the involvement of additional residues in nsP4 function remain to be elucidated.

In this study, we took advantage of the strong structure-function conservation of positive-sense RNA virus RdRps and the power of homology-based modeling to further understand CHIKV nsP4 function. Using the coxsackievirus B3 (CVB3) 3D polymerase, a well-studied model for positive-sense RNA virus polymerases [21,22], we hypothesized that nsP4 residues in close vicinity to the polymerase active site, the GDD motif, and the previously characterized C483 residue could play important roles for polymerase function. To this end, we designed a set of 18 nsP4 variants targeting conserved residues among alphaviruses and investigated how these variants contribute to replication and virion production in mammalian and mosquito cells. Using the full-length virus, we demonstrated that the CHIKV nsP4 palm domain and ring finger participate in host-specific replication and virion production. In addition, we found that CHIKV replication can be enhanced in mammalian cells at low temperature, in contrast to what is seen in mosquito cells, highlighting an influence of the host environment in addition to temperature. Taken together, this study provides novel insight into nsP4 function, describing a key role in alternation between vector and host, and pinpoints important RNA transcription and virion assembly regulatory residues located in the palm domain of the polymerase.

## Results

### Residues in the CHIKV nsP4 palm domain and ring finger subdomain are critical for virus replication in mammalian cells

**Fig 1:**
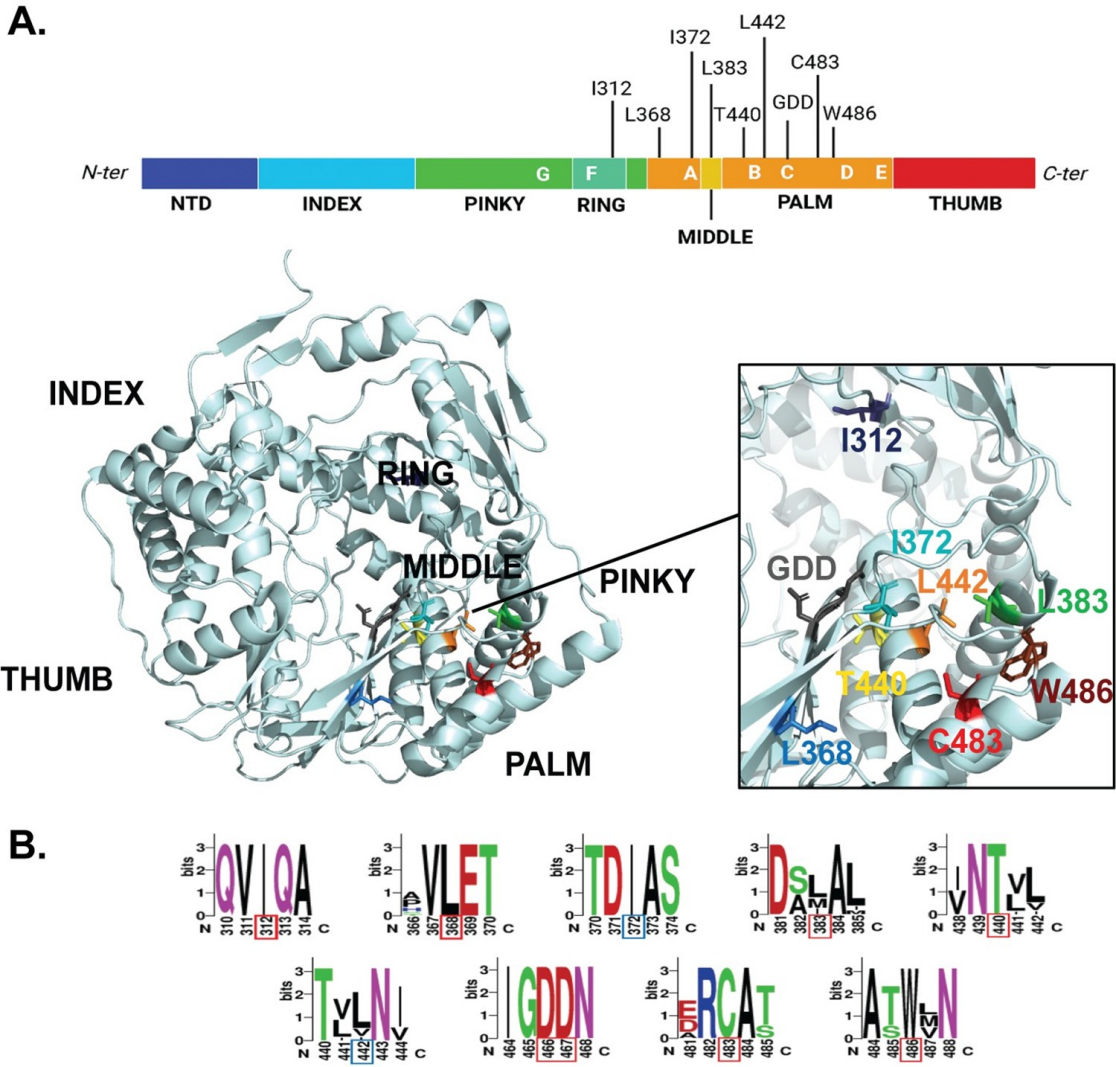
CHIKV nsP4 variant location and conservation. (**A**) Top: CHIKV nsP4 linear structure schematic colored from its N-terminus (dark blue) to its C-terminus (red). Subdomains are indicated below in capital letters and conserved positive-sense RNA virus polymerase motifs are delimited and indicated in white capital letters. Each nsP4 residue is indicated at the top of the protein. Schematic made with BioRender. Bottom: O’nyong’nyong nsP4 3D structure (PDB: 7Y38) annotated with subdomains (black). Mutated residues displayed on nsP4 3D structure with their side chain color-coded as follow from their N-ter to C-ter position in nsP4: I312 (dark blue), L368 (medium blue), I372 (turquoise), L383 (medium green), T440 (yellow), L442 (orange), GDD motif (grey), C483 (red), W486 (brown). (**B**) Conservation of nsP4 residues among alphaviruses generated using the multiple sequence alignment of nsP4 via MUSCLE (EMBL-EMI; see Methods). The height of each stack of letters represents the sequence conservation at the indicated position and the height of each symbol within the stack represents its relative frequency. A patch of five amino acids was selected, with each mutated residue at the center of it framed by a red square (if outside of a conserved motif) or blue (if inside a conserved motif). Each color represents amino acids of similar properties.

Previous studies have shown that the CHIKV nsP4 residue C483, located in the palm domain (**Fig 1A**), plays a key role in polymerase fidelity and host-specific replication [13,17,18]. We hypothesized that other nsP4 residues within this domain would also play important roles for nsP4 function and the CHIKV life cycle. To test this hypothesis, we used homology modeling to design polymerase variants based on the coxsackievirus B3 3D polymerase (CVB3 3D^pol^). A total of 18 CHIKV nsP4 variants were designed by mutating seven amino acids with similar amino-acid substitutions as in the CVB3 3D^pol^ studies (**Fig 1A**) [22,23]. As the crystal structure of the CHIKV polymerase has not been solved yet, we modeled nsP4 variants on the closely related alphavirus O’nyong’nyong nsP4 which is 91% identical at the amino acid level [21] (**Figs 1 and S1**). This panel of CHIKV variants targets nsP4 residues conserved among alphaviruses (**Fig 1B**) including I312, L368 and T440 which correspond to known residues in CVB3 3D^pol^ affecting viral growth [22,23], respectively I176, I230 and S299. In addition, we speculate that the surrounding CHIKV nsP4 residues I372 (Y234 in CVB3 3D^pol^), L383 (W245 in CVB3 3D^pol^), L442 (I301 in CVB3 3D^pol^) and W486 (Y351 in CVB3 3D^pol^) could be involved in polymerase activity due to their respective orientations and locations, either in close vicinity to the GDD motif or to the C483 residue (**Fig 1A**).

As CHIKV replicates in both mammals and mosquitoes in nature, we wanted to elucidate the impact of each nsP4 variant on CHIKV replication in each organism. To do so, we selected as model cell lines, hamster BHK-21 cells, for the mammalian host, and the *Aedes albopictus* C6/36 clone cell line for the mosquito vector. To begin, we tried to rescue each nsP4 variant by transfecting the *in vitro* transcribed full-length CHIKV nsP4 variant RNA into BHK-21 cells. This approach has the advantage to synchronize replication and eliminate any confounding effects of virus entry. At 48 hours post transfection (hpt), we titrated the supernatant by plaque assay on Vero cells to determine infectious viral particle production, quantified extracellular CHIKV genomic RNA (gRNA) by RT-qPCR, and Sanger sequenced the nsP4 variant to address genetic stability.

**Fig 2:**
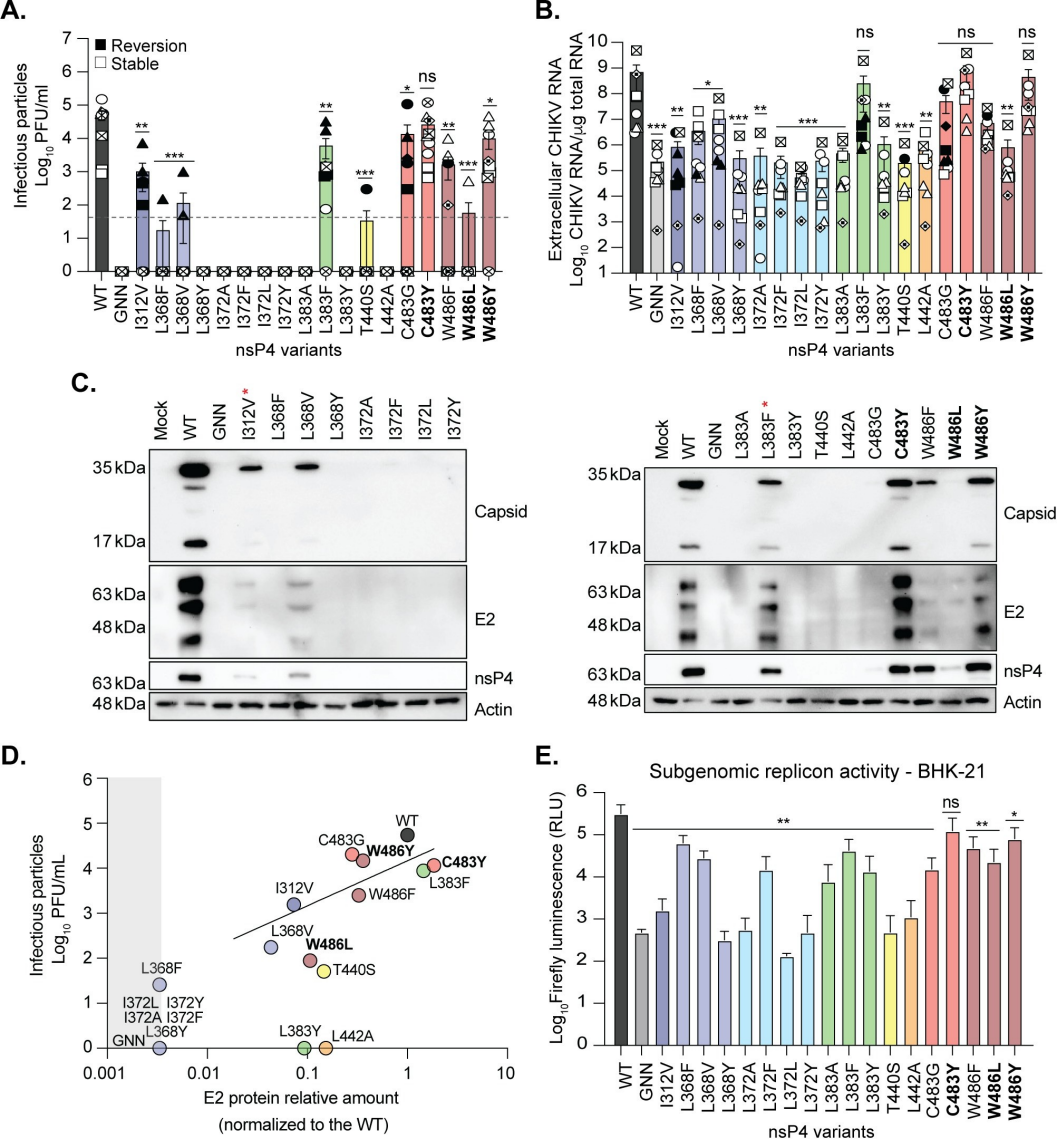
CHIKV nsP4 variant replication is restricted in BHK-21 cells. **A-D.** BHK-21 cells were transfected with *in vitro* transcribed full-length CHIKV RNA of each variant and incubated for 48 hours at 37°C. (**A**) Culture supernatants were harvested at 48 hpt and infectious virus was quantified by plaque assay on Vero cells. The limit of detection of the plaque assay is depicted by the dashed line on both the graph. (**B**) RNA was extracted from culture supernatants and CHIKV genomes were quantified by RT-qPCR. Genetically stable or reverted nsP4 variants are respectively depicted with as an empty symbol or solid black symbol. Bold variants on the *x*-axis are genetically stable. For all panels, each symbol represents an independent biological experiment, in technical transfection duplicate or singlet. A Mann-Whitney *t* test was performed against nsP4 WT on panels A, B and E. Data with statistical significance was labeled as follow: * P<0.05, ** P<0.01, *** P<0.0002, **** P<0.0001. (**C**) Immunoblot of intracellular proteins from transfected BHK-21 cells from panel **A** harvested at 48 hpt for the protein capsid, and E2, nsP4, and actin with molecular weights on the left side as a reference. Red asterisk indicates reversion. Membranes represent one of three independent experiments. Additional experiments can be found in S2 Fig. (**D**) Linear regression analysis of the mean of infectious viral particles and the mean of the amount of the 55 kDa band of the E2 protein relative to its nsP4 WT. A grey shade is highlighting the nsP4 variants behaving like the negative control, nsP4 GNN. Quantification of the protein bands was done with the Biorad Image Lab software. (**E**) BHK-21 cells at 37°C grown in a 96-well plate were transfected with the *in vitro* transcribed subgenomic replicon corresponding to each nsP4 variant for 48 hours. Firefly luminescence was acquired with a luminometer as relative luminescence units. Graph shows the average and SEM of three independent experiments with internal transfection duplicates. A Mann-Whitney *t* test was performed against nsP4 WT and data with statistical significance was labeled as follow: * P<0.05, ** P<0.01, ns = non-significant. Graphs show the average and SEM of six independent experiments with internal transfection duplicates or singlets on panels A to D, and three independent experiments on E.

In BHK-21 cells (**Table 1 and Figs 2 and S9A**), ten of the nsP4 variants produced infectious virus (I312V, L368F, L368V, L383F, T440S, C483G, C483Y, W486F, W486L and W486Y) with each variant, except for C483Y, producing significantly less virus than the WT control. Virus production (**Fig 2A**) was correlated with the presence of extracellular RNA (**Fig 2B**), while nsP4 variants that did not produce infectious virus showed viral RNA levels similar to that of the negative-control GNN active-site variant (**Fig 2B**). However, sequencing of the CHIKV nsP4 region from the rescued variants showed that only three of the nsP4 variants, C483Y, W486L and W486Y, were genetically stable (**Table 1 and Fig 2, bold**). All other rescued nsP4 variants showed either a partial or a total reversion to the WT residue at 48 hpt (**Fig 2**, solid symbols). By looking at the specific codon changes during reversion, we noticed that the majority of revertant viruses presented only one nucleotide change compared to nsP4 WT, with the exception of nsP4 W486F (**Table 1**). This variant reverted by changing two nucleotides, from UUC (F) to UGG (W) with tryptophan being encoded by a unique UGG/TGG codon. Surprisingly, although nsP4 L368F and nsP4 L368V both reverted to WT in BHK-21 cells, the reversion was achieved through two different codons, respectively CUU and UUG (**Table 1**), suggesting a potentially less stringent codon usage when mutating residue 368.

**Table 1:** Rescue of CHIKV nsP4 variants in BHK-21 at 37°C and C6/36 cells at 28°C. The percentage of CHIKV nsP4 variants rescued in six independent experiments in technical transfection singlet or duplicate, their genetic stability, the percentage of revertant amongst the rescued viruses and their reversion genotype is presented.

nsP4 variant	% replicates being rescued		Genetic Stability		% of revertant amongst rescued viruses		Reversion genotype	
	**BHK-21**	**C6/36**	**BHK-21**	**C6/36**	**BHK-21**	**C6/36**	**BHK-21**	**C6/36**
I312V	55	20	Reverted to I	Reverted to I	100	50	GTA (V) to ATA (I)	GTA (V) to ATA (I)
L368F	11	20	Reverted to L	Stable	100	0	TTT (F) to CTT (L)	Stable
L368V	22	20	Reverted to L	Stable	100	0	GTG (V) to TTG (L)	Stable
L368Y	0	0	NR	NR	N/A	N/A	N/A	N/A
I372A	0	0	NR	NR	N/A	N/A	N/A	N/A
I372F	0	0	NR	NR	N/A	N/A	N/A	N/A
I372L	0	0	NR	NR	N/A	N/A	N/A	N/A
I372Y	0	0	NR	NR	N/A	N/A	N/A	N/A
L383A	0	0	NR	NR	N/A	N/A	N/A	N/A
L383F	100	100	Reverted to L	Stable	40	0	TTT (F) to CTT (L)	Stable
L383Y	0	0	NR	NR	N/A	N/A	N/A	N/A
T440S	11	0	Reverted to T	NR	100	N/A	TCA (S) to ACA (T)	N/A
L442A	0	0	NR	NR	N/A	N/A	N/A	N/A
C483G	55	10	Reverted to C	Stable	100	0	GGT (G) to TGT (C)	Stable
C483Y	100	100	Stable	Stable	0	0	Stable	Stable
W486F	44	60	Reverted to W	Stable	25	0	TTC (F) to TGG (W)	Stable
W486L	11	0	Stable	NR	0	N/A	Stable	N/A
W486Y	88	100	Stable	Stable	0	0	Stable	Stable

N/A = not applicable; NR = not rescued

The genetic instability of the nsP4 variants suggests that these variants have a decreased fitness in mammalian cells, resulting in the strong selection of revertant virus over the course of the rescue. In support of this hypothesis, when we immunoblotted the lysates of transfected BHK-21 cells against nsP4, capsid, and E2 (**Fig 2C**), we found that the accumulation of viral proteins was drastically reduced or abolished for the nsP4 variants L368F, L368V, L383A, L383Y, L442A and C483G (**Figs 2C, S2A and S2B**). This reduction in protein accumulation correlated with a reduction in intracellular genomic RNA (**S2C Fig**) and active replication (**S2D Fig**) for many of the nsP4 variants. The presence of the E2 glycoprotein was positively correlated with the release of infectious progeny (**Fig 2D**), with genetic reversion adding to this phenotype observed in the nsP4 variants I312V, L368V, L383F, T440S, C483G and W486F (**Table 1**). For the genetically stable nsP4 variants C483Y and W486Y, we observed equal amounts of nsP4 yet increases or decreases in E2 accumulation compared to WT respectively, while there were minimal changes in extracellular viral particles. These results suggest that subgenomic transcription may be altered in these variants.

Even in the absence of viral proteins for many of the nsP4 variants, we still see genetic reversion, indicating that these variants are still replication-competent which is supported by active replication (**S2C Fig**). To address replicase activity and subgenomic transcription directly, we introduced each variant into a subgenomic replicon expressing firefly luciferase from the subgenomic promoter (**Fig 2E**). We transfected *in vitro* transcribed RNAs into BHK-21 cells and measure luminescence at 48 hpt. We found that several of the variants behaved like the GNN variant (I317V, L368Y, I372A, I372L, I372Y, T440S, and L442A), indicating these variants are significantly impaired in replication in BHK-21 cells. On the other hand, we find another set of variants (L368F, L368V, L372F, L383A, L383F, L383Y, C483G, C483Y, W486F, W486L, and W486Y) that are replication-competent in BHK-21 albeit with lower replication activity ranging from ~2.5 to 40% of the WT replicase activity (**S1 Table**). Taken together, these analyses support that mammalian cells exert a strong selective pressure on the palm and ring subdomains, reducing viral replication, subgenomic transcription, and virion production.

### The CHIKV palm domain is important for virus replication and virion production in mosquito cells

**Fig 3:**
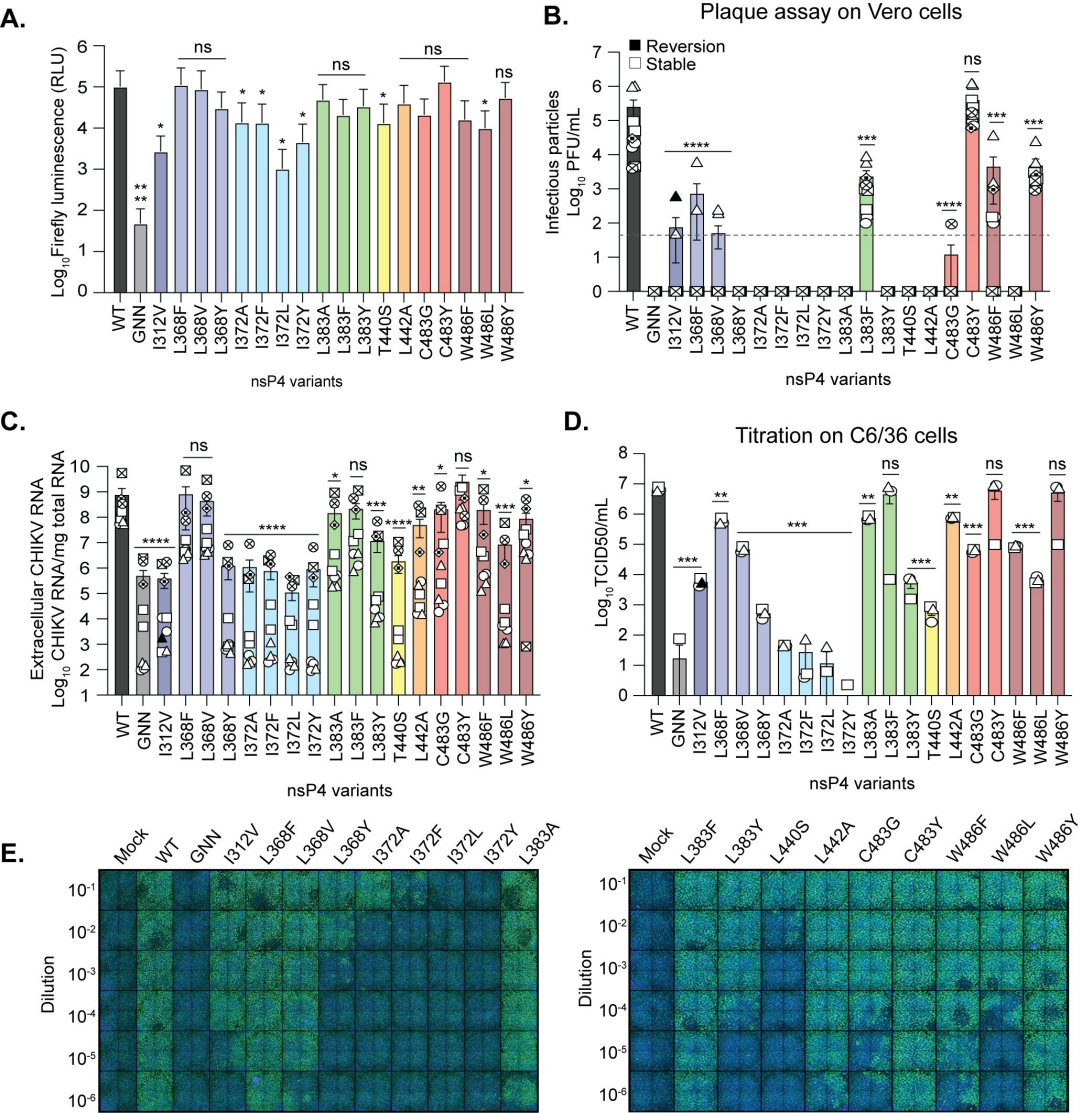
CHIKV nsP4 variants contribute to virion production in mosquito cells. **(A)** C6/36 cells were transfected with the *in vitro* transcribed subgenomic replicon corresponding to each nsP4 variant during 48 hours at 28°C. Firefly luminescence was acquired with a luminometer as relative luminescence units. Graph show the average and SEM of three independent experiments with internal transfection duplicates. A Mann-Whitney *t* test was performed against nsP4 WT and data with statistical significance was labeled as follow: * P < 0.05, **** P<0.0001, ns = non-significant. (**B and C**) C6/36 cells were transfected with *in vitro* transcribed full-length CHIKV RNA of each variant and incubated for 48 hours at 28°C. (**B**) Culture supernatants were harvested at 48 hpt and infectious virus was quantified by plaque assay on Vero cells. The limit of detection of the plaque assay is depicted by the dashed line on the graph. (**C**) RNA was extracted from culture supernatants and CHIKV genomes were quantified by RT-qPCR. For all panels, each symbol represents an independent biological experiment, in technical transfection duplicate or singlet. A Mann-Whitney *t* test was performed against nsP4 WT on panels B and C. Data with statistical significance was labeled as follow: * P<0.05, ** P<0.01, *** P<0.0002, **** P<0.0001. Graphs show the average and SEM of six independent experiments with internal transfection duplicates or singlets on panels B to C. (**D and E**) Supernatants from nsP4 variants produced on mosquito cells in panel B were titrated by a TCID50 assay on mosquito cells at 28°C. C6/36 cells were seeded in a 96-well plate, infected with ten-fold dilutions of supernatant and incubated 3 days at 28°C, fixed and stained with an anti-CHIKV capsid antibody (green) and DAPI (blue). Viral titers were determined based on the lowest dilution in which we could observe at least 2 independent experiments with 50% or more infected cells. A one-way ANOVA test was performed. Data with statistical significance was labeled as follow: * P<0.05, ** P<0.01, *** P<0.0002, **** P<0.0001. Graphs show the average and SEM of three independent experiments.

Given the strong restriction in subgenomic transcription in BHK-21 cells, we wanted to address how each variant contributes to replication in mosquito cells. We transfected C6/36 cells with *in vitro* transcribed subgenomic replicon RNAs containing each variant (**Fig 3A**). In contrast to BHK-21 cells, we found that all nsP4 variants retained replicase activity in C6/36 cells above the GNN variant. However, several of the variants including I312V, I372A/F/L/Y, and W486L had significantly reduced activity compared to the WT replicase. These results show that in C6/36 cells, the nsP4 variants are replication-competent and that subgenomic transcription activity is largely unaffected.

The fact that many of the nsP4 variants had no negative impact on replicase activity allowed us to hypothesize that these variants would have little impact on virus replication. To address virus replication and virion production in mosquito cells, we transfected C6/36 cells with *in vitro* transcribed full-length viral RNA containing each nsP4 variants. We found that eight variants produced infectious virus as tittered on Vero cells (I312V, L368F, L368V, L383F, C483G, C483Y, W486F and W486Y) and sequencing revealed that seven rescued variants were genetically stable, with the exception of nsP4 I312V, which reverted to WT in one replicate (**Table 1 and Fig 3B,** solid symbol). Interestingly, for variants at residue W486, we find infectious virion production more strongly reduced in C6/36 cells compared to BHK-21 cells, suggesting a specific impairment in insect cells. As we observed in BHK-21 cells, extracellular CHIKV genomic RNA correlated with the presence of infectious virions in C6/36 cells for the variants L383F, C483Y, W486F and W486Y (**Fig 3C**). However, the nsP4 variants L368F, L368V, L383A, L383Y, L442A, as well as C483G, often released extracellular viral RNA (**Fig 3C**) in the absence of infectious viral particles (**Fig 3B**), suggesting that these viruses are replicating. It should be noted that no cytopathic effect was observed in these conditions at the moment of the harvest, contrary to nsP4 WT, L383F, C483Y and W486Y variants, minimizing the possibility of intracellular content release upon cell death.

Given the restriction we see in BHK-21 cells (**Fig 2**), we hypothesized that one reason for not obtaining infectious virus from mosquito cells may be due to plaquing on Vero cells where replication may be also restricted. Therefore, we titrated the supernatants from mosquito cells by TCID50/mL assay on C6/36 cells (**Fig 3D and 3E**). Using this assay, we found that the nsP4 variants L383F, C483Y, and W486Y produced viral particles to levels similar to that of WT CHIKV, yet the remainder of the nsP4 variants were producing significantly less viral particles than WT CHIKV. Specifically, the nsP4 variant I312V, L368Y, L383Y, T440S, and W486L were attenuated ~1000-fold while other variants including L368V, L442A, and W486F were attenuated ~100-fold. These results suggest that while replicase activity is largely unchanged (**Fig 3A**), many of these variants have defects in virion production in C6/36 cells. Together, these studies highlight that the nsP4 palm and ring subdomains play critical roles in replication in mammalian and insect cells. Importantly, while many of the nsP4 variants have defects in viral particle production, they are still replication competent and retain subgenomic transcription activity in insect cells.

### Changes in the CHIKV palm subdomain alter structural protein accumulation in mosquito cells

**Fig 4:**
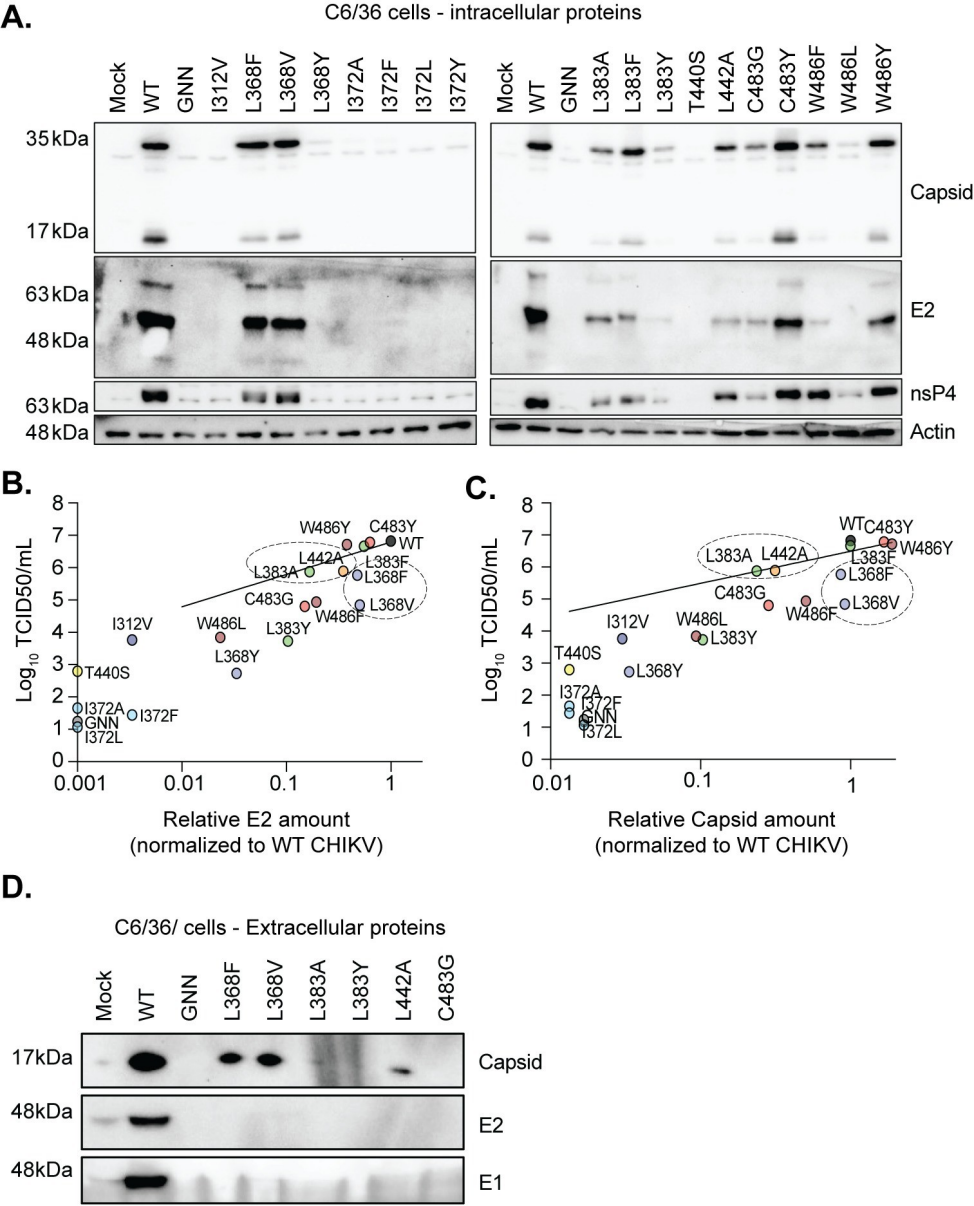
CHIKV nsP4 variants display alterations in protein production and particle secretion. (**A**) C6/36 cells were transfected in a 6-well plate with the nsP4 variants *in vitro* transcribed RNA from Fig 3B and were lysed at 48 hpt for SDS-PAGE and immunoblotted for the CHIKV proteins capsid, E2, nsP4, and the house-keeping gene actin with molecular weights on the left side as a reference. Membranes are representative of three independent experiments. The western blots corresponding to the other independent experiments can be found in S3 Fig. (**B**) A linear regression analysis was performed on infectious viral particles titrated by TCID50 assay on mosquito cells and relative amounts of intracellular E2 (**B**) and capsid (**C**) protein for C6/36 cells from the three independent western blotting presented in (B) and S3 Fig. Dashed circles are pointing to clusters of nsP4 variants of interest. (**D**) Supernatants from C6/36 cells transfected with *in vitro* transcribed full-length CHIKV nsP4 variants were harvested at 48 hpt (corresponding to Fig 3). Extracellular proteins were concentrated with Amicon columns, separated by SDS-PAGE and immunoblotted for capsid (top), E1 (middle), and E2 (bottom). Blot represents one of three independent experiments (S4 Fig).

As we observed a significant discrepancy between viral particles generated from mosquito cells and replicase activity (**Fig 3A, 3D and 3E**), we hypothesized that changes in the nsP4 palm domain may be leading to changes in viral structural protein production. To test this hypothesis, we assessed the production of nsP4 and the structural proteins capsid and E2 by immunoblot at 48 hpt in C6/36 cells from Fig 2 **(Fig 4A).** From these studies, we observed several interesting phenotypes. First, for the nsP4 variants that produced low levels of viral particles (< 10^3^ TCID50/mL; I372A/F/L/Y, L368Y, L383Y, and T440S), we did not detect intracellular viral proteins by western blot (**Fig 4A**). Several of these variants had reductions in replicase activity confirming that these variants are attenuated in mammalian and mosquito cells. Second, the variants I312V, L383Y, and W486L produced slightly more viral particles (~10^4^−10^5^ TCID50/mL), yet while we could detect viral proteins from L383Y and W486L, we could not detect any viral proteins from the I312V variant in three independent replicates (**Figs 3, S3A and S3B**), suggesting that maybe there are other secreted vesicles carrying viral genetic material. Next, the nsP4 variants L368V, C483G, and W486F produce ~10^5^ TCID50/mL which correlates with detectable viral proteins. However, we see dramatic differences in the amount of nsP4, E2, and capsid between L368V and C483G even though they are producing the same amount of viral particles (**Fig 4B and 4C**). The variants L368F, L383A, and L442A also produce a significant amount of viral particles, and while the L368F variant has high accumulation of viral proteins, the L383A and L442A variants accumulate much less viral proteins, yet still produce similar amounts of viral particles (**Fig 4B and 4C**) and have replicase function similar to WT CHIKV (**Fig 3A**). Finally, the variants L383F, C483Y, and W486Y produce equal amounts of viral particles to WT CHIKV, yet again the L383F variant accumulates significantly less viral proteins. Together, these results suggest that while replicase function is similar to WT CHIKV, there may be changes in subgenomic RNA translation and/or protein accumulation. These changes in nsP4 may increase the efficiency of virus production, able to produce high levels of viral particles from less protein in the case of the L383A variant, or for the case of L368F/V, these viruses may have defects in virus infectivity or spread.

To address secreted viral particle production directly, supernatants from C6/36 cells transfected with these nsP4 variants at 48 hpt in Fig 3 were concentrated with Amicon Ultra centrifugal filters to address the presence of capsid, E1 and E2 by immunoblotting (**Figs 4D** and **S4**). Interestingly, we detected only small amounts of capsid protein for the nsP4 variants L368F, L368V and L442A, alongside nsP4 WT (**Fig 4D**). Moreover, no E1 or E2 could be recovered in this specific experiment. Only a very small amount of E2 protein appeared for nsP4 L368V in another independent experiment (**S4 Fig**) but no capsid or E1 protein was recovered for these remaining biological replicates, except for nsP4 WT. We found it interesting that we are unable to detect viral proteins from samples with high levels of viral particles (L368F, L383A, and L442A). While this may be a detection issue it will be interesting to investigate these particles further, as we cannot exclude the release of any sort of vesicles containing viral material, infectious or not, in the supernatant.

**Fig 5:**
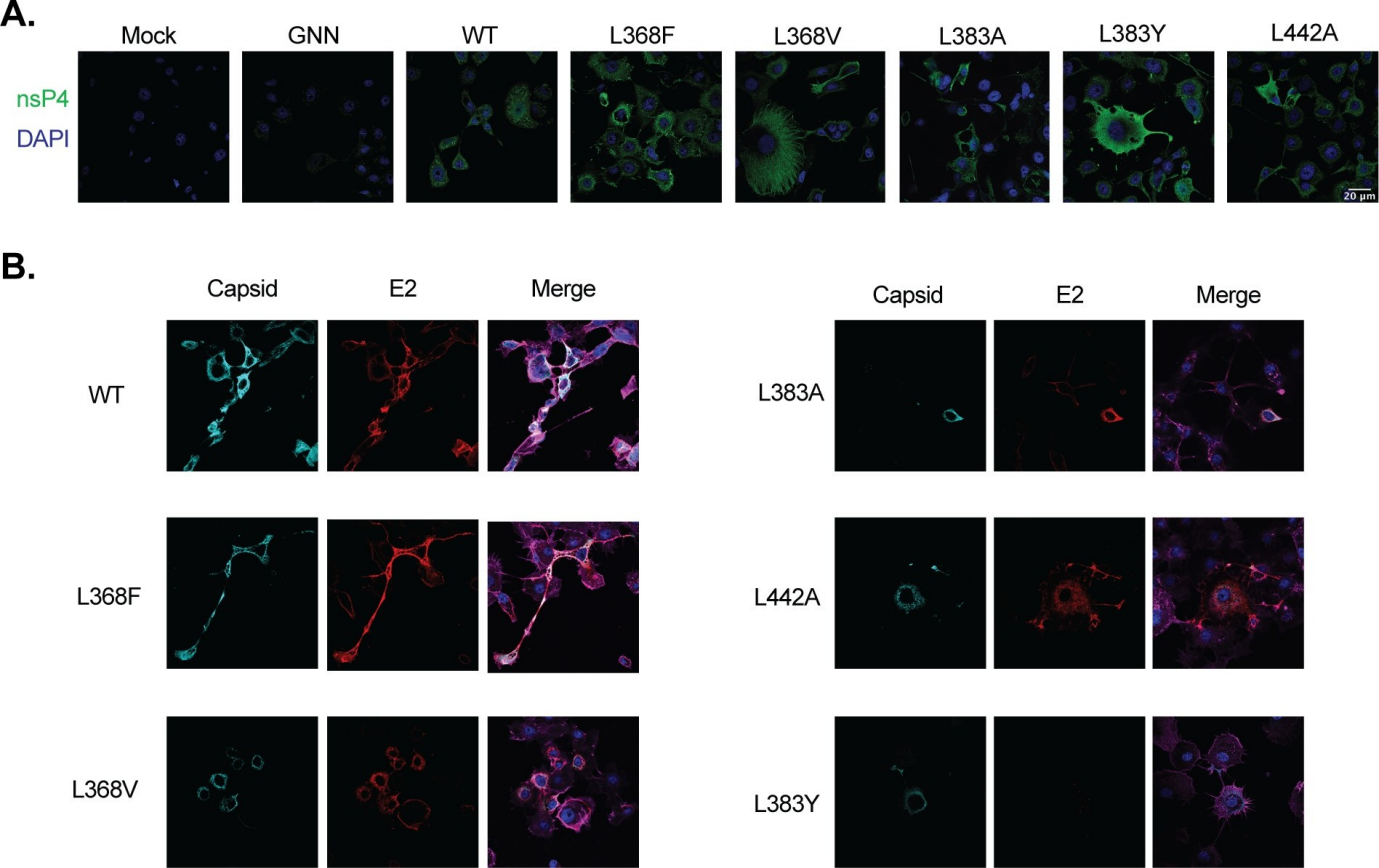
Nonstructural and structural protein localization of nsP4 variants. C6/36 cells were seeded onto coverslips and transfected with *in vitro* transcribed full-length CHIKV nsP4 variants then incubated for 24 hours at 28°C. Cells were fixed and stained for (**A**) nsP4 (green) and DAPI (blue) and (**B**) capsid (teal), E2 (red), actin (magenta) and DAPI (blue) and images acquired on a confocal microscope. Images are representative of two independent experiments for all panels. Scale bar: 20 μm. Images of negative control mock and nsP4 GNN transfected cells as well as additional nsP4 variant images can be found in S6–S8 Figs.

Finally, to address any impact of the nsP4 mutation on viral protein subcellular localization, we investigated the localization of nsP4 and the structural proteins capsid and E2 (**Figs 5 and S6–S8**). We performed immunofluorescence at 24 hpt on the same selection of nsP4 variants as in Fig 4D, staining for nsP4 (**Figs 5A and S6**) and capsid, E2, and actin (**Figs 5B, S7 and S8**). For all the attenuated nsP4 variants, L368F, L368V, L383A, L383Y and L442A, intracellular polymerase expression was similar to nsP4 WT (**Fig 5A**), yet did observe differences in gross cell morphology. Looking then at structural proteins and actin expression (**Fig 5B**), we noted several interesting observations. First, nsP4 WT, L368F, L368V, and L383A all expressed capsid and E2 proteins, even if with some difference in intensity correlating with the western blot (**Figs 5B, S2A and S2B**). On the other hand, at 24 hpt, the nsP4 variant L383Y do not express detectable E2 (**Figs 5B and S6B**), an observation that correlates with the absence of viral proteins being released at 48 hpt (**Fig 4E**). Finally, we observed the expression of E2 but very little of capsid for the nsP4 variant L442A at 24 hpt (**Figs 5B and S6B**), whereas both proteins are expressed at 48 hpt (**Figs 4B, S2A and S2B**). In addition, while for WT CHIKV we found capsid and E2 to colocalize in distinct concentrated foci, capsid and E2 of the L368V, L383A, L442A, and L383Y were more dispersed and granular. Of note, it seems that at least the nsP4 variant L368F, as nsP4 WT, was able to produce intercellular extensions that have been described as actin and tubulin positive and of more than 10 μm long [24] (see scale bar on **Figs 4A and S6B**) while other variants did not yield these structures. These CHIKV-induced extensions have been recently shown to be required for cell-to-cell viral spread and to participate in CHIKV’s ability to escape circulating neutralizing antibodies [25]. In addition, they require the presence of both capsid and E2 proteins for their formation. Looking further, we noted that the F-actin network was disorganized for the nsP4 variants L383A and L442A, and its expression less pronounced for the nsP4 variant L383Y (**Figs 5A and S6B**). Importantly, many viruses are known to manipulate the actin network at their advantage [26] and actin has been described as crucial for viral structural protein transport to the assembly sites and virus assembly in general [27–30]. Together, these results suggest that despite a comparable subgenomic RNA transcription as WT CHIKV in mosquito cells, several of the nsP4 variants including L368V, L383A, L383Y, and L442A likely present a defect in viral protein accumulation or translocation, consequently impairing the ratio of capsid and E2 required for a proper virion assembly.

### Low temperature rescues CHIKV nsP4 palm domain variant replication in mammalian cells

**Fig 6:**
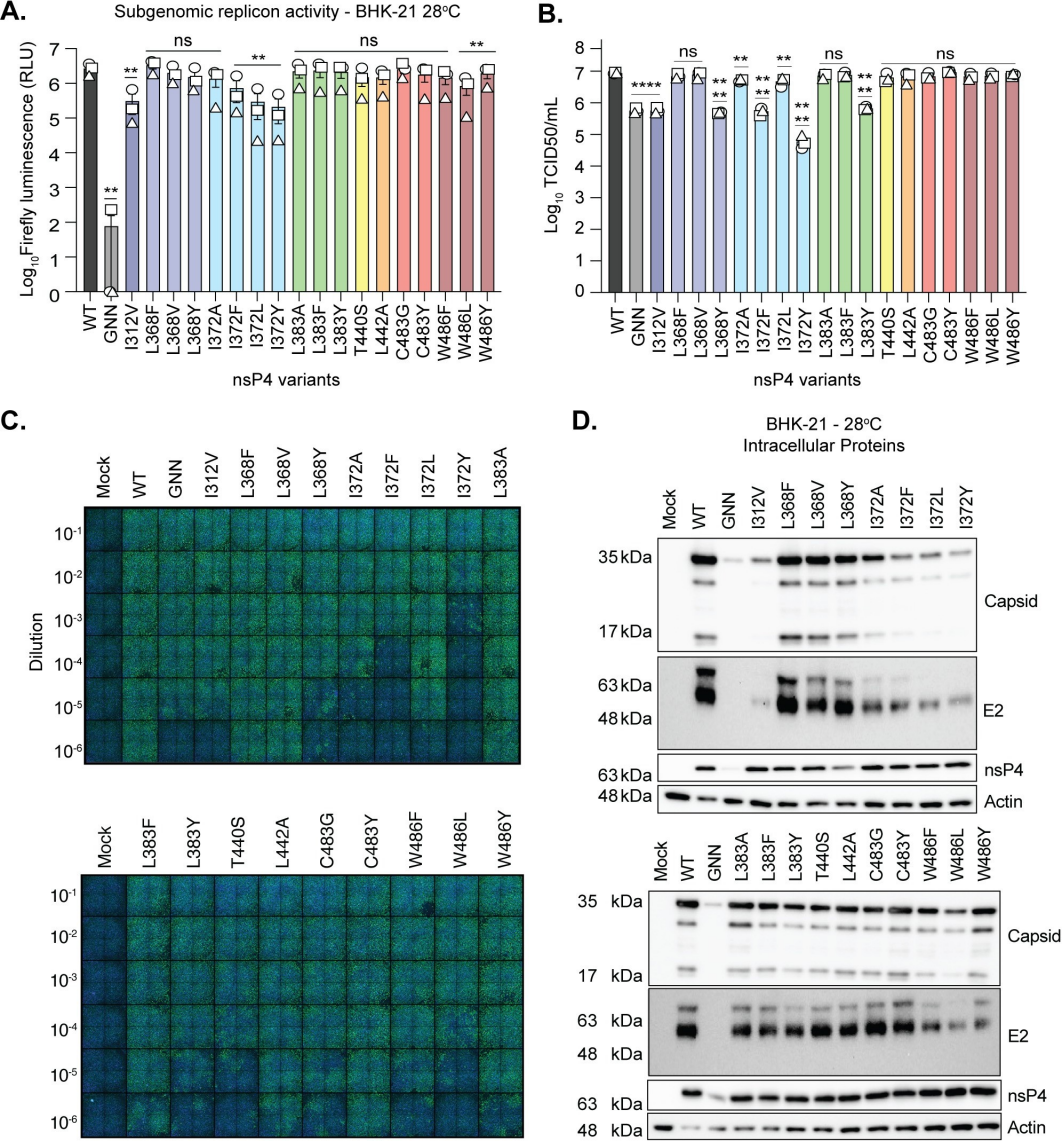
Low temperature rescues CHIKV nsP4 variant replication. (**A**) BHK-21 cells grown at 28°C were transfected with the *in vitro* transcribed subgenomic replicon corresponding to each nsP4 variant for 48 hours. Firefly luminescence was acquired with a luminometer as relative luminescence units. Graph shows the average and SEM of three independent experiments with internal transfection duplicates. A Mann-Whitney *t* test was performed against nsP4 WT and data with statistical significance was labeled as follow: * P<0.05, ** P<0.01. **(B and C)** BHK-21 cells adapted to grow at 28°C were transfected with *in vitro* transcribed full-length CHIKV RNA of each variant and incubated for 72 hours. Viral titers were quantified by TCID50 on C6/36 cells at 28°C. Viral titers were determined based on the lowest dilution in which we could observe at least 2 independent experiments with 50% or more infected cells. A one-way ANOVA test was performed. Data with statistical significance was labeled as follow: * P<0.05, ** P<0.01, *** P<0.0002, **** P<0.0001. Graphs show the average and SEM of three independent experiments. (**D**) BHK-21 cells from panel B were lysed, separated by SDS-PAGE, and immunoblotted for the CHIKV proteins capsid, E2, nsP4, and the house-keeping gene actin with molecular weights on the left side as a reference. Membranes are representative of two independent experiments. The western blots corresponding to the other independent experiments can be found in S5 Fig.

Arboviruses are unique in that they replicate in mammalian organisms, with a body temperature around 37°C, and in arthropod organisms, with a body temperature around 28°C [31,32], making temperature an important parameter to consider. As several alphavirus temperature-sensitive mutants have been described in the literature [33–44], one reason for the restriction in BHK-21 and Vero cells may be due to elevated temperature. To test this hypothesis, we transfected the *in vitro* transcribed subgenomic replicon RNA containing each nsP4 variants into BHK-21 cells adapted to 28°C (**Fig 6A**) and measured firefly luminescence at 48 hpt. In BHK-21 cells at 28°C, subgenomic RNA transcription was enhanced significantly both in the WT replicase as well as for each variant (compare Figs 2E and 6A). However, the nsP4 variants at I312, I372 and W486 still showed a reduction in subgenomic transcription similar to what was seen for the replicon and full-length virus at 37°C and in C6/36 cells at 28°C (**Figs 2 and 3**), supporting that these residues play key roles in CHIKV replication in both hosts.

We then asked whether temperature could rescue full-length virus replication (**Fig 6B-D**). We transfected BHK-21 cells adapted to 28°C with *in vitro* transcribed full-length viral RNA coding for the nsP4 variants and harvested virus containing supernatants and cells at 72 hpt, as infection is expected to progress slower than at 37°C [45,46]. When we quantified viral particles in the supernatants by TCID50/mL assay on mosquito cells (**Fig 6B and 6C**), we could now rescue viral particles from all the nsP4 variants to levels higher than at 37°C and in C6/36 cells at 28°C. The variants I312V, L368Y, I372F, I372Y and L383Y produced less viral particles than WT or other nsP4 variants, indicating that these variants still have defects in the virus life cycle even at low temperature. Interestingly, we also obtained high levels of infectious virus from the GNN active site variant (**Fig 6B and 6C**). In line with this observation, it has previously been noted that the GNN polymerase is a weakened polymerase and not a dead polymerase rigorously speaking, contrary to a GAA nsP4 [9], thus emphasizing that a low temperature in BHK-21 cells may enhance basal RNA polymerase activity in general. Importantly, we did not see an equivalent enhancement of the GNN variant in the context of the replicon (**Fig 6A**), suggesting that in the context of the live virus these variants may be genetically unstable leading to particle production.

We then hypothesized that viral protein production would also be restored at low temperature. When we addressed viral nonstructural and structural protein accumulation at low temperature in BHK-21 cells, we found that many of the variants restored protein production at 28°C (**Fig 6D**), in sharp contrast to BHK-21 cells grown at 37°C (**Fig 3**) and C6/36 cells grown at 28°C (**Fig 4**). In addition, we now found viral proteins in the GNN variant, confirming the enhanced replication. However, even in the presence of high viral titers in the supernatant comparable to the WT virus, several of the nsP4 variants at positions I312, I372 and W486 showed reduced protein accumulation, confirming that these variants have defects even at 28°C. Of interest, the nsP4 variants from the residues I312 and I372 in BHK-21 cells at 37°C (**Fig 2A**) did not produce viral proteins, but each of them did to some extent in mammalian cells incubated at 28°C (**Fig 6D**). Taken together, these results show that adapting BHK-21 cells to 28°C can enhance WT and nsP4 variant replication. However, given that in C6/36 cells at 28°C we still see significant defects in virion and protein production not seen in BHK-21 cells at 28°C, we hypothesize adapting BHK-21 cells to lower temperatures induces cell-specific changes that drive these phenotypes.

### Virus derived from low temperature adapted BHK-21 cells have altered viral protein compositions and infectivity

**Fig 7:**
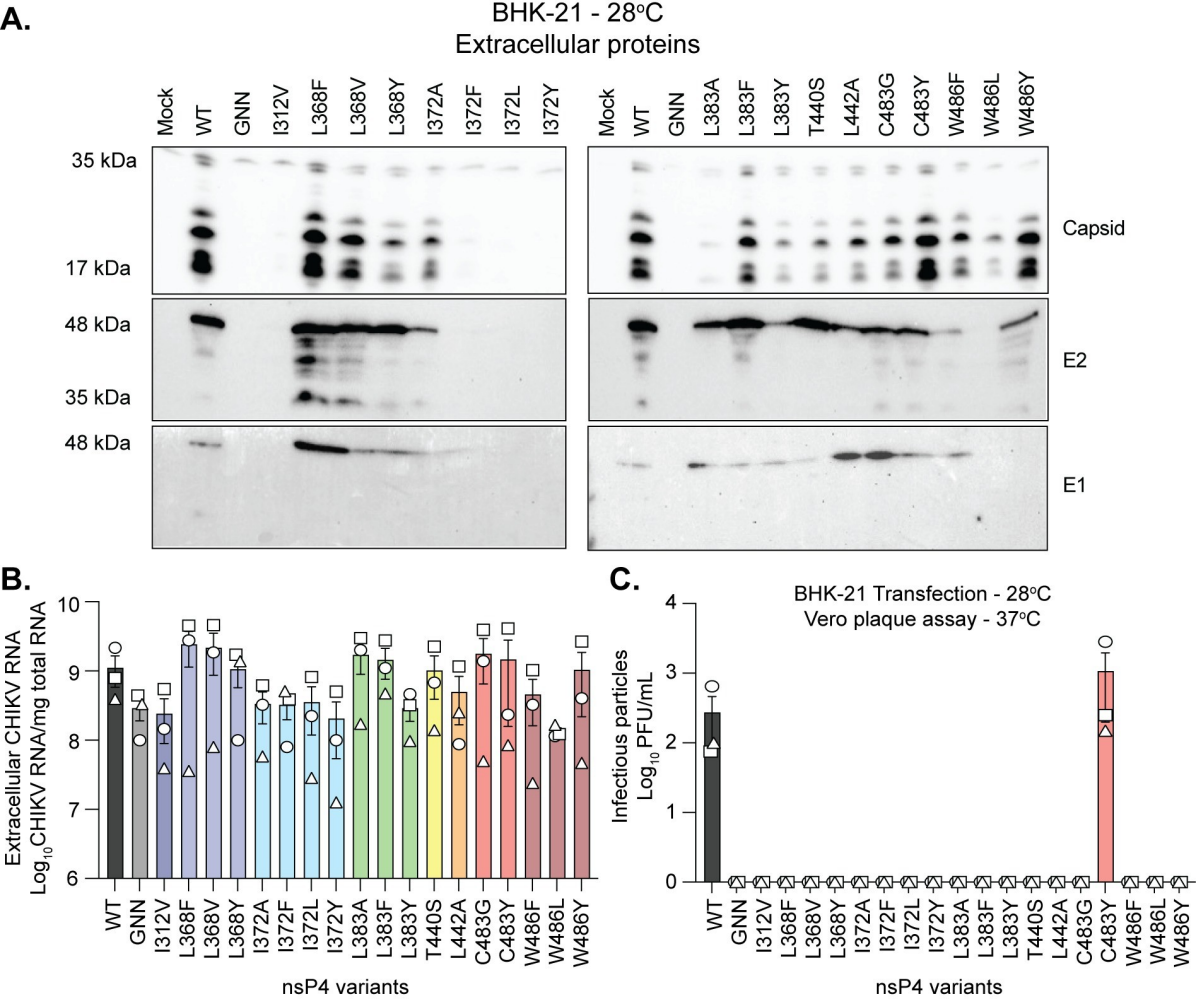
Virions produced in BHK-21 cells at low temperature have altered protein levels and infectivity. (**A**) Extracellular proteins from supernatants from Fig 6B were concentrated with Amicon columns, separated by SDS-PAGE, and immunoblotted for the presence of the proteins capsid, E2, and E1. Membranes are representative of two independent experiments. The western blots corresponding to the other independent experiment can be found in S5 Fig. (**B**) RNA was extracted from culture supernatants and CHIKV genomes were quantified by RT-qPCR. (**C**) Culture supernatants were harvested at 72hpt, and infectious virus was quantified by plaque assay on Vero cells at 37°C. For all panels, data represent the mean and SEM and each symbol represents an independent biological experiment, in technical transfection singlet, with solid symbols showing the nsP4 variants that reverted to the WT residue and clear symbols the variants that did not revert.

In mosquito cells grown at 28°C we were able to identify viral particles via TCID50, however we were unable to find viral proteins in the supernatant (**Fig 4D**). One explanation for this phenotype could be technical issue due to the limit of detection of the immunoblot. Therefore, we wondered whether we could retrieve structural proteins from supernatants of transfected BHK-21 cells at 28°C. Thus, we immunoblotted for the structural proteins E1, E2 and capsid on concentrated supernatants at 72 hpt from BHK-21 cells adapted to grow at 28°C (**Fig 7A**). We found that the supernatants of WT CHIKV grown in BHK-21 cells at 28°C contained multiple species of capsid protein as well as low levels of E1 compared to E2 (**Fig 7A**). Nevertheless, we could retrieve all three structural proteins, E1, E2 and capsid in supernatants from the majority of the nsP4 variants except for the nsP4 variants GNN, I312V, I372F,L, and Y (**Fig 7A**). Here, we were able to observe viral structural proteins from the variants L368Y, I372A, and L383Y which yielded ~10^6^ TCID50/mL of viral particles from BHK-21 cells at 28°C, similar to the titers observed from L368V, L383A, and L442A from C6/36 cells at 28°C, where we could not detect viral proteins in the supernatant. In addition, we also observed that for the GNN and I312V variants, where we detect high levels of viral particles by TCID50, we were unable to detect viral structural proteins in the supernatant while we were able to detect proteins from the variants L368Y and L383Y. These results may suggest the presence of secreted vesicles containing viral genetic material rather than or in addition to true infectious virions. Going further, we asked whether these viral particles contained viral genomes. We thus determined the presence of gRNA in the supernatants by RT-qPCR (**Fig 7B**). Interestingly, we observed high levels of extracellular gRNA for the nsP4 variants L368F/V/Y, L383A, T440S, C483G, C483Y and W486Y (**Fig 7B**). This suggested that the viral structures produced by these nsP4 variants were containing gRNA.

Finally, as many of the nsP4 variants still replicate and form plaques at 37°C (**Fig 2B**), we wanted to quantify infectious virus in the supernatant of nsP4 variants produced in BHK-21 cells adapted to 28°C on Vero cells at 37°C by plaque assay. Interestingly, we were only able to rescue the nsP4 WT and nsP4 C483Y viruses (**Fig 7C**), contrary to mosquito cells in which the nsP4 variants I312V, L368F, L368V, L383F, C483G, C483Y, W486F and W486Y were rescued on Vero cells at 37°C (**Fig 3B**). These results were surprising, as using the subgenomic replicon system several nsP4 variants were replicating well (**Fig 3E, nsP4 L368F/V, L383F, W486F/Y**) and produced infectious particles in BHK-21 cells at 37°C (**Fig 2**). Moreover, the titers of the WT and nsP4 C483Y variant were significantly reduced when the virus was produced in BHK-21 cells at 28°C and then titrated at 37°C (**Fig 7C**) compared to virus both produced and titrated at 37°C (**Fig 2B**). Having in mind that subgenomic RNA synthesis in mammalian cells at 28°C was enhanced dramatically (**Fig 6**), the rescue of only the WT and C483Y nsP4 variant pinpoints that low temperature may be impacting the infectivity of the virions or altering the cell biology, such as the ability to produce exosomes containing viral material for example. Lastly, to evaluate whether the lower temperature provided an advantage to nsP4 C483Y variant over WT CHIKV, we performed a replication kinetic by titrating the supernatant of transfected BHK-21 cells by plaque assay at 24, 48 and 72 hpt (**S5C Fig**). Interestingly, the nsP4 WT virus was only rescued starting at 48 hpt, contrary to the nsP4 C483Y virus producing infectious particles at 24 hpt. Thus, the high-fidelity CHIKV nsP4 variant possesses a fitness advantage at 28°C compared to WT CHIKV. Taken together, we conclude from these analyses that overall CHIKV sgRNA transcription is enhanced in mammalian cells grown at 28°C and adapting the cells to this temperature rescues the sgRNA synthesis of the nsP4 variants. Thus, highlighting an important interplay between temperature and cell biology on polymerase activity and providing insight into the observed restriction of mammalian cells to CHIKV polymerase variants replication.

## Discussion

Alphaviruses are significant human health threats, yet there are no or limited therapeutic strategies available. One potential broad-spectrum antiviral target is nsP4, the highly conserved RNA-dependent RNA polymerase, highlighting the need to understand how the alphavirus polymerase functions. In this study, we took advantage of the CVB3 3D^pol^, a well-characterized model for positive-sense RNA virus polymerase study, and the predictive power of homology-modeling to unravel unknown key CHIKV polymerase activity determinants. With this objective, we predicted a set of variants from seven conserved CHIKV nsP4 palm domain residues based on the CVB3 3D^pol^ structure. We hypothesize that these residues may play critical roles in nsP4 replication and function.

**Fig 8:**
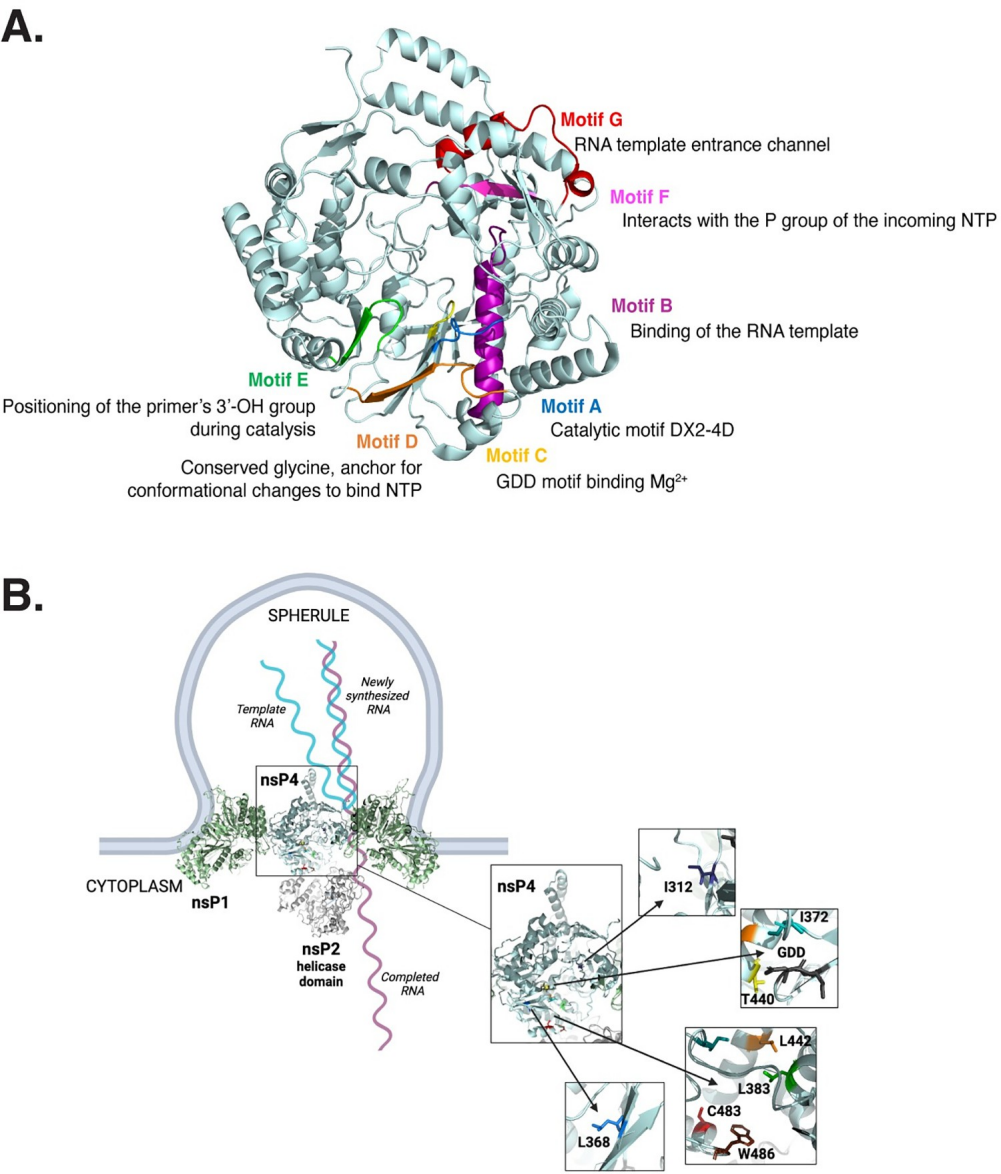
nsP4 variants are close to crucial conserved enzymatic activity motifs and the helicase domain of nsP2 in the context of the core replicase complex. **(A)** nsP4 3D structure (PDB: 7Y38) showing in color each of the conserved positive-sense RNA-dependent RNA polymerase motifs and their respective function. Motif A is depicted in blue, motif B in purple, motif C in yellow, motif D in orange, motif E in green, motif F in pink, and motif G in red. (**B**) nsP4 in the context of the core replicase complex (PDB: 7Y38). A zoom on nsP4 and the different pockets corresponding to the nsP4 variants is shown. The residues L383, C483 and W486 are located at the interface between nsP4 and nsP2. Schematic made with Biorender.

When we tried to rescue each nsP4 variant in mammalian cells, we found that only the variants C483Y, W486L and W486Y remained genetically stable, suggesting a strong selective pressure in mammalian cells. Interestingly, while the CHIKV nsP4 variants I312V and L368F were not genetically stable in mammalian cells, the corresponding CVB3 3D^pol^ variants were stable in human cells [22], highlighting differences between viruses or cell types. On the contrary, in mosquito cells, all rescued nsP4 variants except the nsP4 I312V variant were genetically stable. The I312 residue is located close to the polymerase ring finger (**Figs 1A and 8**). The finger subdomains are highly flexible subdomains involved in conformational changes during polymerization that maintain the active closed structure of the catalytic site and are required for RNA template binding [9,47]. Given that the I312V variant was unstable in both mammalian and mosquito cells, it suggests a potential key role for the ring finger in both species.

In line with the genetic instability in BHK-21 cells, we observed a strong restriction in nsP4 variant replication both at the level of infectious virus and intracellular protein production. One explanation for these phenotypes may be that these variants are temperature-sensitive in BHK-21 cells and restricted by growth at 37°C. To disentangle the effect of temperature from the cellular environment, we adapted BHK-21 cells to grow at 28°C and show that replication of all the CHIKV nsP4 variants, including WT CHIKV and the GNN variant, was drastically enhanced. These results are interesting in light of our data in C6/36 cells, which were also grown at 28°C. We hypothesized that if temperature is the parameter influencing the phenotypes in BHK-21 cells, then virus replication and virion production should also be rescued in C6/36 which are grown at 28°C. This hypothesis is supported by a recent preprint aimed at understanding the impact of the alphavirus unique domain (AUD) of nsP3 on CHIKV replication [48]. Here, Guo and Harris characterized several AUD mutants and found that replication of these mutants was significantly reduced in mammalian cells comparing to WT CHIKV, yet viral replication was not affected in mosquito cells grown at 28°C. As temperature seemed to be an obvious factor to investigate, they grew mouse myoblasts cells at 28°C and demonstrated that viral replication and protein translation was enhanced at this temperature, similar to our results. However, we do not see this same trend with these nsP4 variants. While the variants may be sensitive to temperature in BHK-21 cells, virion and protein production are restricted in C6/36 cells at 28°C. These results highlight the importance of these nsP4 domains and the host in the viral life cycle.

Our temperature-sensitive findings in BHK-21 cells are intriguing and the fact that WT CHIKV as well as the GNN active-site variant also have enhanced replication and virion production in BHK-21 cells grown at 28°C is exciting. In addition, we find differences in protein species and accumulation in virions grown at 28°C as well as lower infectious viral titers for WT CHIKV. These findings suggest that adapting BHK-21 cells to 28°C may significantly alter the general cell biology including cellular metabolism, RNA transcription, and/or host factors which could impact virus biology. Biochemically, temperature could affect the fluidity of the plasma membrane [49,50], along with the nature of the lipids composing it. Furthermore, cholesterol, notably enriched in lipid rafts, has been demonstrated to play a crucial role in entry, replication and exit of alphaviruses [51–53], such as CHIKV [54,55], likely by stabilizing the membrane invaginations required for each of these replication steps. Aside of membrane fluidity, temperature could also affect the viral replication rate [46,56,57] and the virion stability [58–60]. Indeed, Guo and Harris show that at 28°C, there were both an increase in the number of replication sites in the cell and an enhanced recruitment of stress granule proteins into these active replication sites compared to 37°C (54). Therefore, temperature will be a powerful tool to study virus and cell biology in the future.

There are many temperature-sensitive alphavirus variants, also known as *ts* mutants, described in the literature. *ts* mutants have been mainly investigated for Sindbis virus (SINV) and Semliki Forest virus (SFV). In these studies, temperature sensitivity was used as tool to study lethal mutants and gain insight into viral replication and biology. SFV *ts* mutants were discovered following a N-methyl-N’ nitro-N-nitrosoguanidine treatment, a chemical mutagenesis agent. Several of these mutants were not able to synthesize viral RNA at a restrictive temperature in chick embryo fibroblasts [34] or the equivalent in BHK-21 cells [35,36] while several *ts* mutants had mutations in the protease or helicase domain of nsP2 [36,37], mutations in nsP1 [35], or in nsP4 [44], thus affecting either subgenomic RNA transcription, minus-strand RNA and/or genomic RNA synthesis in infected cells. In parallel, a lot of work has been done to characterize *ts* mutants of SINV Heat-Resistant strain (HR strain) [40]. These *ts* mutant viruses were characterized by an impairment in viral RNA synthesis, either in the complementary minus-strand RNA or the subgenomic RNA synthesis, when put at a restrictive temperature in chick embryo fibroblasts [38–40,44]. Interestingly, these studies demonstrate that alphavirus replication and more specifically RNA synthesis is abrogated at a restrictive temperature generally higher than the physiological temperature of the mammalian cells used.

In these published studies, several *ts* mutants presented substitutions in nsP4, G153Q for SINV *ts*6, G324Q for SINV *ts*110 and Q93R for SINV *ts*118 [39,44] and A242V for SFV *ts*13. Putting these residues into the active CHIKV replication complex structure [19], nsP4 residues from SINV *ts*6, *ts*118 and SFV *ts*13 are directly facing the nsP1 molecules forming the dodecameric ring sitting at the base of each spherule. Interestingly, residue 93 in CHIKV nsP4 is facing the same pocket as does the residue I312. This observation brings light to the fact that these *ts* mutants may not form active replication complexes at 40°C but retain their activity if the complexes were able to form at 30°C (permissive temperature), before to be shifted to a restrictive temperature. The only exception is *ts*6 for which, even if the replication complexes are successfully formed at 30°C, they cannot function at 40°C, likely because the temperature sensitivity of this *ts* mutant is at the level of transcription elongation rather than formation of the replication complexes *per se*, via interaction with nsP1 molecules.

Additionally, nsP3 is known to sequester stress granule proteins, such as G3BPs [61,62]. By doing so, nsP3 stimulates subgenomic RNA transcription and the downstream viral proteins translation thus indirectly affecting viral assembly [63]. This underlines that temperature could also indirectly influence viral replication, not only by destabilizing replication complexes or RNA elongation, as shown in SINV and SFV *ts* mutant studies, but also by impacting the availability and/or recruitment of host-co-factors. The residue L368, by its location in the open groove between nsP2 helicase domain and the molecules of nsP1 inserted in the plasma membrane (**Fig 8B**), could potentially interact with either a viral protein, such as nsP3 as suggested by the subtomogram map of the replication complex generated by Tan et al [19], or a host partner. Linking observations by Guo and Harris and our study, we could hypothesize that in the context of CHIKV replication complex, point mutation in nsP4 could impact either protein-protein or protein-RNA interactions at a restrictive temperature (37°C), whereas replication complexes would have primarily evolved to have efficient formation and smooth interactions at a permissive temperature (28°C). Even if there are still few supporting data on insect-specific alphaviruses, it is thought that arboviruses evolved from insect-specific viruses that acquired the ability to expand their host range to vertebrates, such as birds or mammals [64–66], highlighting the importance of being able to replicate at disparate temperatures.

In C6/36 cells which are grown at 28°C, we find that many of the variants have defects in protein accumulation and virion production. Specifically, variants at residue I372, produced low to no viral particles in the context of the full-length virus. Looking at the polymerase structure, I372 is located in the conserved motif A and in immediate proximity to the catalytic aspartic acid residue at position 371 involved in the coordination of the magnesium cations during catalysis (**Figs 1B, 8A and 8B**) [6,9,20]. This observation underlines the strong evolutionary constraints taking place in close vicinity to the polymerase active site, an idea supported by the Protein Contact Atlas predicted interactions that indicates strong atomic level interactions between the I372 residue and the catalytic residues D371 and D466 [67]. Importantly, motif A has been described to align with motif C upon binding of the correct NTP, initiating the closed and active RdRp conformation required for polymerization [47], thus emphasizing the pressure on this specific region of the protein (**Fig 8A**). The results with the nsP4 I372 variants are particularly interesting when we compare the full-length virus to the subgenomic replicon. Using the subgenomic replicon system, the nsP4 I372 variants are replication-competent in C6/36 and BHK-21 cells grow at 28°C, suggesting there are minor defects in RNA synthesis. However, we find no protein accumulation or virions produced. One explanation for this phenotype could be that in the full-length virus, the sgRNA itself or the structural proteins may be important for proper polymerase function, and when it is replaced by a reporter construct, these constraints are lifted. These observations may indicate that using a subgenomic replicon reporter assay or even a reporter virus may not directly reflect work with the full-length native virus.

In addition to variants at residue I372, we found that in C6/36 cells replicon activity was largely unaffected for many of the variants while virion and protein production were heavily impacted. Residue L368 is intriguing as it is located just before the conserved motif A in the palm domain but is also quite structurally isolated from both the catalytic site pocket and the C483 pocket (**Fig 8B**). Residue L442, on the other hand, is located inside motif B, involved in the binding of the RNA template [47], allowing us to speculate that even a slight change in amino acid side chain length, as in between a leucine and an alanine, has an impact on the correct positioning of the template in the polymerase, given the reduction in protein production and viral progeny observed in this study. We also observed by immunofluorescence that the nsP4 variants L383A and L442A, despite expressing structural proteins, displayed an altered actin distribution in mosquito cells. This observation is of particular interest as a wide variety of viruses are known to manipulate the actin network at their advantage, either for entry, replication, assembly, or egress [26,28–30,68]. While few studies have been done on alphaviruses in general [69–71], it seems that CHIKV may rely on the actin network and its modulation to produce viral progeny [72–74].

One striking observation was that even in the presence of viral particles by TCID50, we found little intracellular or extracellular viral proteins. One hypothesis for this phenotype may be that mosquito cells are releasing exosomes containing viral genetic material, potentially infectious, as it has already been described following Dengue virus infection in C6/36 cells [75]. In consequence, we could only observe it with a sensitive assay such as a TCID50 assay. Previous work has shown that using the low-fidelity SINV polymerase C482G variant, the analog of the C483G variant for CHIKV nsP4, recombines at a higher rate than WT virus and produces large amounts of defective interfering particles (DIPs) [76]. DIPs are sub-viral particles that generally contain truncations of the viral genome, rendering it unable to undergo a full replication cycle by interfering with viral replication [77]. Of note, it has been demonstrated that temperature indirectly affects the production of DVGs in dual-host positive sense RNA viruses by altering the processivity of the RdRp, as observed with Influenza A virus [78,79], but also the stability of secondary RNA structures important for viral replication, as shown for West Nile virus [80]. Therefore, it may be likely that changes in the nsP4 palm subdomain could influence vesicle and DI secretion, potentially explaining our results and even the reduced viral plaquing titers from BHK-21 cells grown at 28°C. Further work is required to disentangle the contribution of these parameters on CHIKV polymerase activity across hosts. In future studies, it will be interesting to investigate the impact of the nsP4 variants on the genetic diversity of the progeny and so indirectly assess their replication fidelity and DI production.

The idea of nsP4 being involved in virus production is intriguing. Putting back the nsP4 variants studied in this work in the context of the replication complex solved by Tan *et al* (**Fig 8B**), residues L368, L383, and L442 locate to the cytoplasmic side of the spherule near the interface of the helicase domain of nsP2 with nsP4 (19). While this structure lacks nsP3, we could hypothesize that replication complexes and both their viral as well as host components are not totally isolated from other replication events happening either in the cytoplasm, such as translation of the structural proteins, or at the plasma membrane, such virion assembly and budding. Despite providing evidence for it in this study, a proximity between viral replication complexes and viral assembly sites has been evoked [81–83] and could facilitate packaging of gRNA by an increased accessibility. Interestingly, gRNA is selectively packaged in the nucleocapsid whereas present in small amounts in the cell during replication compared to the sgRNA. Viral packaging requires both RNA-protein interactions and protein-protein interactions involving respectively, and chronologically, gRNA-capsid, capsid/capsid then capsid/E2 interactions [84–86]. Work with VEEV and SFV has suggested that nsP2 could be involved in packaging [87] and the nsP2 protein has been shown to regulate viral assembly of infectious virions and tightly interacts with the helix I of capsid protein [88] and nsP4 [13,19,89]. Additionally, the CHIKV nsP3 AUD was also described to be involved in virus assembly through the production of sgRNA [63]. Outside of alphaviruses, replication and assembly have been demonstrated to be strongly connected. Specific mutations in the flavivirus non-structural proteins NS2A and NS3 block infectious particle production [90–93]. NS2A has particularly been established as key during the assembly process by recruiting both the new viral RNA and the structural proteins C, prM and E. In addition, the assembly sites are located in the same continuous endoplasmic reticulum-derived membrane network as the replication sites [94]. Finally, a spatial coupling between replication and assembly has been demonstrated for SARS-Cov2 in the double-membrane vesicles it reshapes from the endoplasmic reticulum [95]. Thus, we can speculate based on these studies on other positive-strand RNA viruses that the nonstructural and structural proteins of alphaviruses are likely cooperating more than previously thought and that mutating the polymerase could impact in cascade other downstream replication steps.

Finally, another way that the polymerase could influence downstream steps upon infection is simply by increasing the local concentration of specific species of viral RNA. In this line, a study showed that the capsid protein from Flock house virus, a bipartite positive strand RNA virus, translated from a nonreplicating RNA produced particles containing cellular RNAs, and not only viral RNA, as did a capsid protein translated from a replicating RNA [96]. Thus, we could think that in the case of a point mutation in nsP4, even slightly altering its polymerase function like the synthesis of sgRNA, an imbalance is created in the cell towards an increased abundance of cellular RNAs, thus impacting the infectivity of the newly generated virions. Therefore, it will be of interest to investigate the presence of nucleocapsid cores and budding particles by cryo-electronic microscopy in the context of the abortive nsP4 variants. Lastly, a ratio of 1:1 between capsid and E2 is required for a proper virion assembly [97,98]. This is an important point that should be investigated as we could observe in this study differences in the amount of these structural proteins to one another for a given nsP4 variant.

While our work describes important domains for CHIKV nsP4 function and replication through alternate hosts, it possesses several limitations. First, the cell lines we selected for this study are particularly permissive to CHIKV infection and more broadly to arboviral infections as BHK-21 cells are defective at producing interferon [99] and C6/36 cells do not have a competent RNAi pathway due to a defect in Dicer-2 [100,101]. The simplicity of *in vitro* models and the absence of a functional RNAi pathway in C6/36 cells [100], may remove a selective pressure on viral infection that is found *in vivo*. Therefore, looking at nsP4 variant replication in RNAi- or interferon-competent cells, like U4.4 cells, also derived from *Aedes albopictus* mosquitoes, or human fibroblasts will give us insight into the impact of this immune selective pressure on viral replication itself. Finally, as our targeted approach focuses on a specific panel of mutants, we only have a partial view of functional residues in the polymerase. Investigating the impact of the genetically stable nsP4 mutations on the molecular biology of the polymerase and the genetic diversity of the viral progeny would be particularly interesting and expanding outside of the palm domain and ring finger will be key to have a bigger picture of how nsP4 functions.

In conclusion, these studies investigate how discrete domains of the CHIKV polymerase function in the complete alphavirus life cycle. We identified novel determinants required for polymerase RNA transcription and protein and virion production. Furthermore, it underlines the differential selective pressures encountered by the polymerase during replication. As perspectives, for a more integrated and comprehensive overview, *in vivo* models could be used to provide insight on the attenuation of the rescuable nsP4 variants in their natural environment. Finally, as we highlighted important host- and temperature- specific pressures, it would be interesting to see if our observations on CHIKV nsP4 could be expanded to other alphaviruses, notably to alphaviruses known to circulate in *Culex spp*. species (SINV, RRV or VEEV) or to have birds as a natural reservoir (SINV, RRV) [102,103].

## Materials and methods

### Cell lines

Baby hamster kidney cells (BHK-21 CCL-10, ATCC) were grown in Dulbecco’s Modified Eagle Medium (DMEM, Corning, #10-017-CV) supplemented with 10% fetal bovine serum (FBS, Atlanta Biologicals), 10 mM HEPES (Gibco, #15630080), and 1% nonessential amino acids (MEM-NEAA, Gibco, #11140050). Vero cells (CCL-81, ATCC) were grown in DMEM supplemented with 10% newborn calf serum (NBCS, Sigma). All mammalian cell lines were maintained at 37°C under controlled 5% CO_2._ *Aedes albopictus* cell clone C6/36 (CRL-1660, ATCC) was grown in Leibovitz’s medium (L-15, Corning, #10-045-CV) supplemented with 10% FBS, 1% tryptose phosphate broth (Gibco, #18050039) and 1% MEM-NEAA, Gibco, # 11140050) at 28°C with 5% CO_2_. To test the nsP4 variants for temperature sensitivity, BHK-21 cells were adapted to 28°C for three passages. Each cell line was tested monthly for mycoplasma using the Lookout Mycoplasma PCR detection kit (Sigma-Aldrich, # MP0035) and confirmed mycoplasma free. BHK-21 and C6/36 cells of passage 10 or less were used for every experiment (or alternatively less than 1-month in culture).

### Homology modeling

An initial model for the CHIKV nsP4 structure was developed by a combination of homology modeling and threading onto the coxsackievirus B3 polymerase structure (PDB: 3DDK), using the programs I-TASSER [104], Swiss-model [105], and Phyre [106]. This approach provided a reasonable model for the conserved active site motifs in the palm domain that allowed us to identify residues likely to generate fidelity-modulating variants based on our prior work with coxsackievirus B3 polymerase [23,107]. Residues were chosen based on their proximity to the pre-existing C483Y variant [17] or having locations likely to impact the palm domain-based movement of motif A that underlies the active site closure mechanism of positive-strand RNA virus polymerases [34,75]. The locations of these residues were later validated when the structures of Sindbis (SINV) and Ross River virus (RRV) and ONNV [9,19] polymerases were solved.

### Molecular biology and plasmid mutagenesis

The chikungunya virus (CHIKV) replicon (piCRES-Rep) expressing firefly luciferase under the control of the subgenomic promoter was a gift from Dr. Andres Merits (University of Tartu). To introduce the nsP4 mutations, first a cloning vector was made by digesting piCRES-Rep with EcoRV restriction endonuclease, removing the restriction fragment, and re-ligating the plasmid. Using the cloning vector (piCRES-EcoRV), the nsP4 variants were introduced by site-directed mutagenesis using Phusion polymerase following the manufacturer’s instructions with the primers in **Table 2**. After PCR, the plasmid region between the PshAI and AvrII restriction sites was Sanger sequenced to confirm each mutation. Finally, the PshAI/AvrII restriction fragment from the cloning vector was subcloned into piCRES-Rep to generate working plasmids.

**Table 2:** Mutagenesis primers used in this study (mutated nucleotides are in bold).

CHIKV nsP4 variant	Forward Primer	Reverse Primer
I312V	ACCTAAGGTGCAGGTT**GTA**CAGGCGGCTGAACCCT	AGGGTTCAGCCGCCTG**TAC**AACCTGCACCTTAGGT
L368F	GCCAGGAGACACTGTT**TTC**GAAACGGACATAGCCT	AGGCTATGTCCGTTTC**GAA**AACAGTGTCTCCTGGC
L368V	GCCAGGAGACACTGTT**GTG**GAAACGGACATAGCCT	AGGCTATGTCCGTTTC**CAC**AACAGTGTCTCCTGGC
L368Y	GCCAGGAGACACTGTT**TAC**GAAACGGACATAGCCT	AGGCTATGTCCGTTTC**GTA**AACAGTGTCTCCTGGC
I372A	TGTTTTGGAAACGGAC**GCA**GCCTCCTTTGATAAGA	TCTTATCAAAGGAGGC**TGC**GTCCGTTTCCAAAACA
I372F	TGTTTTGGAAACGGAC**TTC**GCCTCCTTTGATAAGA	TCTTATCAAAGGAGGC**GAA**GTCCGTTTCCAAAACA
I372L	TGTTTTGGAAACGGAC**CTA**GCCTCCTTTGATAAGA	TCTTATCAAAGGAGGC**TAG**GTCCGTTTCCAAAACA
I372Y	TGTTTTGGAAACGGAC**TAC**GCCTCCTTTGATAAGA	TCTTATCAAAGGAGGC**GTA**GTCCGTTTCCAAAACA
L383A	GAGCCAAGATGATTCA**GCT**GCGCTTACTGCTTTGA	TCAAAGCAGTAAGCGC**AGC**TGAATCATCTTGGCTC
L383F	GAGCCAAGATGATTCA**TTT**GCGCTTACTGCTTTGA	TCAAAGCAGTAAGCGC**AAA**TGAATCATCTTGGCTC
L383Y	GAGCCAAGATGATTCA**TAT**GCGCTTACTGCTTTGA	TCAAAGCAGTAAGCGC**ATA**TGAATCATCTTGGCTC
T440S	AACTCTGTTCGTCAAC**TCA**TTGTTAAACATCACCA	TGGTGATGTTTAACAA**TGA**GTTGACGAACAGAGTT
L442A	GTTCGTCAACACATTG**GCA**AACATCACCATCGCCA	TGGCGATGGTGATGTT**TGC**CAATGTGTTGACGAAC
C483G	GAATTGATGGCAGCCAGA**GGT**GCCACTTGGATGAA	TTCATCCAAGTGGC**ACC**TCTGGCTGCCATCAATTC
C483Y	GAATTGATGGCAGCCAGA**TAT**GCCACTTGGATGAA	TTCATCCAAGTGGC**ATA**CTCTGGCTGCCATCAATTC
W486F	AGCCAGATGTGCCACT**TTC**ATGAACATGGAAGTGA	TCACTTCCATGTTCAT**GAA**AGTGGCACATCTGGCT
W486L	AGCCAGATGTGCCACT**TTG**ATGAACATGGAAGTG	TCACTTCCATGTTCAT**CAA**AGTGGCACATCTGGCT
W486Y	AGCCAGATGTGCCACT**TAC**ATGAACATGGAAGTGA	TCACTTCCATGTTCAT**GTA**AGTGGCACATCTGGCT

To generate full-length wild-type (WT) CHIKV (Strain 06–049; AM258994) and nsP4 variants, a modified CHIKV infectious clone containing an AvrII restriction site at the 3’ end of the subgenomic promoter was used (pCHIK-FLIC-AvrII) [108]. The nsP4 variants were introduced into a small cloning vector via site-directed mutagenesis using the primers in **Table 2** and Sanger sequenced to confirm the variant. The AgeI/AvrII restriction fragment was then subcloned from each nsP4 variant into pCHIK-FLIC-AvrII and each plasmid was Sanger sequenced to confirm the nsP4 variants.

For all studies, 10 μg of each plasmid was linearization overnight with NotI restriction enzyme. The next day, the linearized product was purified by phenol:chloroform extraction and ethanol precipitation and re-suspended in RNAse free water. The purified product was then *in vitro* transcribed using the mMessage mMachine SP6 kit (Invitrogen, # AM1340) following manufacturer’s instructions. *In vitro* transcribed RNA was purified by phenol:chloroform extraction and ethanol precipitation, diluted into RNAse free water at 1 μg/μL and aliquoted before storage at -80°C.

### Biosafety

All experiments with WT CHIKV and CHIKV nsP4 variants were performed in a biosafety level 3 (BSL3) laboratory at NYU Grossman School of Medicine (New York, NY USA).

### Virus rescue experiments

BHK-21 cells, either at 37°C or 28°C, and C6/36 cells were seeded in duplicate in a 6-well plate at a respective concentration of 300,000 cells/well or 600,000 cells/well to be 70–80% confluent the next day for transfection. 24 hours later, 2.5 μg of each RNA was transfected into each well with Lipofectamine MessengerMAX Transfection Reagent (Invitrogen, # LMRNA001) at a respective v/v ratio of 1:2 (RNA:Lipofectamine). After liposomes formation, each mix was added dropwise on top of the corresponding well. 24 hours after transfection, cell culture medium was replaced with fresh complete media until the end of the experiment. Supernatant and cells were harvested 48 hours post-transfection to perform plaque assay, intra- and extra-cellular RT-qPCR, Western Blotting, and deep-sequencing.

### Plaque assay

Infectious virus was quantified by titration on Vero cells at 37°C. Briefly, Vero cells were seeded in 12-well plates at a concentration of 350,000 cells/well. The next day, ten-fold dilutions of each supernatant were made in DMEM and 200 μL of virus was added to each well. Cells were incubated during 1 hour at 37°C. After virus adsorption, 1.5 mL of pre-warmed plaquing medium made of DMEM-2% NBCS-1% antibiotic-antimycotic (Invitrogen, # 15240062) was added per well. Plates were incubated for three days at 37°C and fixed with 1.5 mL of 4% formalin per well (Fisher Scientific, # SF1004) for 1 hour at room temperature. After fixation, formalin and agarose plugs were removed, wells were stained with crystal violet (Sigma-Aldrich, # HT90132) for 15 mins, and plates were washed in diluted bleach (10% Clorox bleach, Fisher Scientific, # 50371500). Viral titers were quantified by counting plaques on the lowest countable dilution.

### TCID50/mL

To address the presence of infectious viral particles in the supernatant from transfected mosquito cells and from transfected mammalian cells grown at 28°C, we quantified the number of infected cells by intracellular staining on C6/36 cells. Briefly, C6/36 cells were seeded in 96-well plate at 50,000 cells/well to be 70–80% confluent the next day for infection. 24 hours later, ten-fold dilutions of each supernatant were made in L15 2% FBS 1% tryptose phosphate broth 1% MEM-NEAA, and 100 μL of virus was added to each well. Plates were incubated for three days at 28°C, supernatant was removed, and wells were fixed with 100 μL of 4% paraformaldehyde (Formaldehyde Aqueous Solution EM grade, 32%, Liquid Electron Microscopy Sciences, #15700) for 1 hour at room temperature. After fixation, wells were washed twice with 100 μL of Perm/Wash solution (BD Perm/Wash buffer, #BDB554723, Fisher Scientific) and permeabilized for 10 min at room temperature with 50 μL of 0.25% Triton X-100 (Triton X-100, Fisher BioReagents, # BP151100) per well, followed by 1 hour of blocking in 100 μL of phosphate buffered saline (PBS; Corning, #21031CV) containing 0.2% bovine serum albumin (BSA) and 0.05% Saponin at room temperature. After blocking, 50 μL of anti-CHIKV capsid antibody (rabbit, CHIK-122, a gift from Dr. Andres Merits) diluted to 1:2000 in blocking buffer was added to incubate overnight at 4°C. The next day, wells were washed four times with Perm/Wash solution and incubated for 1 hour at room temperature with 50 μL of secondary antibody goat anti-rabbit-Alexa 488 diluted to the 1:2000 in blocking buffer. Following incubation, wells were washed three times in Perm/Wash solution and 50 μL of DAPI diluted to the 1:1000 in PBS was added for 30 min, before washing the plates two times with PBS. 100 μL of PBS was ultimately added to each well. The number of infected cells was quantified on the CellInsight CX7 LZR High Content Scanning System (Thermo Scientific). Viral titers were determined based on the lowest ten-fold dilution in which we could observe 50% or more infected cells.

### RNA extraction and RT-qPCR

For extracellular RNA quantification, supernatant was harvested from the producing cells in a 6-well plate format and spun down for 3 mins at 1200 rpm to pellet any cell debris. 200 μL of cleared supernatant was then mixed with 200 μL of Trizol reagent and RNA extracted using the PureLink RNA mini kit (Invitrogen, # 12183025) following the manufacturer’s instructions. For intracellular RNA quantification, RNA extraction was performed using Trizol reagent (Invitrogen, # 15596026) following the manufacturer’s instructions. Briefly, after cell lysis into Trizol, 1/5^th^ of the total volume of chloroform was added in each sample and vortexed for 10 secs, followed by 5 mins incubation at room temperature. Samples were then centrifuged at 12,000 x g for 15 mins at 4°C to allow for phase separation. Following the spin, the upper aqueous phase was transferred to a new tube containing 500 μL of molecular grade isopropanol (absolute isopropanol molecular grade, Fisher Scientific, # 67-63-0) and 1 μL of Glycoblue co-precipitant (Invitrogen, # AM9515). RNA was precipitated overnight at -20°C. The next day, the samples were vortexed and then centrifuged at 12,000 x g for 20 mins at 4°C. Supernatant was removed, and the RNA pellet was washed two times with 700 μL of 75% molecular grade ethanol (Absolute ethanol molecular grade, Fisher Scientific, # BP28184). Finally, ethanol was removed and the pellet was resuspended in 100 μL of RNAse free water. Samples were normalized to 100 ng/μl with RNAse free water and stored at -80°C.

For RT-qPCR, the number of viral genomes/mL was quantified in a transparent 96-well plate by TaqMan RT-qPCR on the extracted RNA (see above). The TaqMan RNA-to-Ct One-Step-RT-PCR kit (Applied Biosystems, # 4392938) was used along with CHIKV primers and a FAM-probe targeting the nonstructural protein 4 (CHIKV Fwd IOLqPCR-6856F: 5’-TCACTCCCTGCTGGACTTGATAGA –3’ / CHIKV Rev IOLqPCR-6981R: 5’-TTGACGAACAGAGTTAGGAACATACC –3’/ CHIKV probe IOLqPCR6919-FAM: 5’-AGGTACGCGCTTCAAGTTCGGCG -3’). The following mix was done for each reaction: 12.5 μL of Taqman RT-qPCR mix 2X, 6.25 μL of RNAse-free water, 0.25 μL of CHIKV Fwd IOLqPCR-6856F, 0.25 μL of CHIKV Rev IOLqPCR-6981R, 0.15 μL of CHIKV probe IOLqPCR6919-FAM, 0.63 μL of Taqman enzyme mix and 5 μL of sample. A standard curve was generated for each plate using ten-fold dilutions of *in vitro* transcribed WT CHIKV RNA. Each experimental sample and standard curve dilution was run in technical duplicates. The plate was then sealed, centrifuged briefly and ran on a QuantiStudio 3 real-time machine (Applied Biosystems). The following program was then used: 48°C for 30 minutes, followed by 10 minutes at 95°C and amplification was done over 40 cycles of 95°C for 15 seconds and 60°C for 1 minute. The amount of CHIKV RNA molecules per mL of sample was determined via the associated standard curve.

To determine if active replication was occurring in the cell, the relative amount of genomic (nsP4) or subgenomic (E1) CHIKV RNA was quantified by Sybr Green RT-qPCR using the Power SYBR Green PCR Master Mix (Thermo Scientific, # 4367659). First, cDNA synthesis was performed using the Maxima H Minus First Strand cDNA Synthesis Kit (Applied Biosystems, # K1652). 1 μL of random primers and 1 μL of dNTPs were mixed with 13 μL of extracted RNA per reaction in a PCR tube. Samples were first subjected to a PCR step at 65°C for 5 mins then chilled on ice and spun before adding a mix of 4 μL of RT buffer and 1 μL of reverse transcriptase enzyme (Maxima H RT) per reaction. The samples were then put at 25°C for 10 mins to allow annealing of the random hexamer primers, followed by 30 mins at 50°C for synthesis of the cDNA. Finally, the reaction is terminated by heating at 85°C for 5 mins. Generated cDNA was then stored at -20°C until use. For the Sybr Green qPCR, the following primers were used: CHIKV nsP4 Fwd IOLqPCR-6856F: 5’- TCACTCCCTGCTGGACTTGATAGA -3’; CHIKV nsP4 Rev IOLqPCR-6981R: 5’-TTGACGAACAGAGTTAGGAACATACC -3’/ CHIKV E1 Fwd 10865F: 5’- TCGACGCGCCCTCTTTAA -3’; CHIKV E1 Rev 10991R: 5’- ATCGAATGCACCGCACACT -3’ / Mouse 18S Fwd: 5’- GTAACCCGTTGAACCCCATT -3’; Mouse 18S Rev: 5’- CCATCCAATCGGTAGTAGCG -3’/ *Aedes* 18S Fwd: 5’- GGTCGGCGCGGTCGTAGTGTGG -3’; *Aedes* 18S Rev: 5’- TCCTGGTGGTGCCCTTCCGTCAAT -3’. Briefly, an 18 uL reaction per well of 96-well plate was done as following: 10 uL of SybrGreen Master Mix, 0.1 uL of 100 uM Fwd primer, 0.1 uL of 100 uM Rev primer and 7.8 uL of RNAse-free water. 2 uL of cDNA was then added per well. The plate was sealed, centrifuged briefly, and ran on a QuantiStudio 3 real-time machine (Applied Biosystems). Amplification was done over 40 cycles of 95°C for 15 seconds and 60°C for 1 minute. The relative expression of each target gene was normalized to their respective 18S expression (either Mouse 18S for BHK-21 cells or *Aedes* 18S for C6/36 cells) using the ΔCt method. The ratio of subgenomic (E1) to genomic (nsP4) CHIKV RNA was then calculated as a proxy for active viral replication.

### Replicon luciferase assay

BHK-21 cells, C6/36 cells, and BHK-21 cells grown at 28°C were seeded in duplicate in a 96-well plate at a concentration of 25,000 cells/well to be 70–80% confluent the next day for transfection. 24 hours later, a total of 0.1 μg of each subgenomic replicon *in vitro* transcribed RNA per well was transfected with Lipofectamine MessengerMAX transfection reagent as described above. After liposome formation, each mix was added dropwise on top of the corresponding wells and cells incubated at 37°C or 28°C. 48 hours later, supernatant was removed and Firefly luminescence was recorded using the Dual luciferase reporter assay system (Promega, # E1910). In brief, the cells were lysed in 20 μL of Passive Lysis Buffer per well. After re-suspension of the lysates, 10 μL of lysate per well was transferred in a white 96-well plate. 30 μL of Luciferase Assay Reagent II per well was finally added right before acquisition of Firefly luminescence (10 sec measurement time) on a luminometer (BioTek Synergy HTX Multimode reader, Agilent Technologies). The luminescence was expressed in Relative Luminescence Units (RLU).

### Immunofluorescence staining and confocal microscopy

C6/36 cells were seeded in a 24-well plate on 12 mm round coverslips (Round German coverslip 12 mm, Bellco Glass, # 1943-10012A) at a concentration of 150,000 cells/well to be 70–80% confluent the next day for transfection. 24 hours later, cells were transfected with 0.5 μg of *in vitro* transcribed RNA as described above. 24 hours later, the wells were fixed by adding 10% paraformaldehyde at a v/v ratio for 1 hour at room temperature. After fixation, the supernatant was removed and cells were washed 3 times with PBS before proceeding to the immunofluorescence staining. Free aldehydes were neutralized with 50 mM ammonium chloride (NH_4_Cl) for 10 mins at room temperature. After several PBS washes, cells were permeabilized 0.1% Triton X100 for 15 mins and then blocked with 2% BSA for 30 mins at room temperature. Cells were then incubated via inversion of the coverslip on 30 μL of primary antibodies diluted at 1:300 in the 2% BSA solution for 1 hour at room temperature in a humid chamber. After incubation, coverslips were put back in the plate, washed several times with PBS and then incubated with fluorophore-conjugated secondary antibodies, diluted at 1:1000, for 45 mins. The following primary antibodies were used: anti-CHIKV nsP4 (rabbit, a gift provided by Dr. Andres Merits at the University of Tartu, Estonia), anti-CHIKV E2 (mouse, the following reagent was obtained through BEI Resources, NIAID, NIH: Monoclonal Anti-Chikungunya Virus E2 Envelope Glycoprotein, Clone CHIK-263 (produced *in vitro*), NR-44003), anti-CHIKV Capsid (rabbit, CHIK-122) and anti-Phalloidin-A488 (F-actin, ActinGreen 488 Ready Probes reagent, Invitrogen, #R37110). Coupled antibodies were incubated after the non-coupled ones, if used in the staining. As secondary antibody, either a goat anti-rabbit IgG-Alexa 488 (Invitrogen, #A-11008), a donkey anti-mouse IgG-Alexa 488 (Invitrogen, #A-31570), a goat anti-rabbit Alexa 647 (Invitrogen, #A-21244) or a donkey anti-mouse Alexa 555 (Invitrogen, #A-31570) were used. Finally, cells were labeled with DAPI and coverslips mounted in ProLong Diamond Antifade (Molecular Probes, #P36965) on slides (SuperFrost End Slide 1 mm thick, Electron Microscopy Sciences, #71867–01). After drying, coverslips were sealed using transparent nail polish. Slides were then stored at -80°C until acquisition on a Zeiss LSM880 confocal microscope (Carl Zeiss). Images were finally analyzed with the Fiji (Image J) software.

### Western blotting

For western blotting, the wells of each 6-well plate were scrapped on ice in PBS. Cells were then centrifuged at 5,000 rpm for 5 mins at 4°C. PBS was removed and 60 μL of lysis buffer (1X TBS, 1 mM EDTA, 1% Triton X-100, 1% protease inhibitor) added into each tube. After 1 hour of incubation at 4°C, 60 μL of 2x Laemmli buffer with 10% 2-β-mercaptoethanol was added per tube. Lysates were then heat-denatured for 10 mins at 95°C and spun for 5 mins at 10,000 x g before storage at -20°C. For SDS-PAGE, samples were thawed at room temperature and 10 μL of sample per well was loaded on a 10% acrylamide gel for nsP4 and E1, and E2 and a 12% acrylamide gel for Capsid. After 50 mins of migration, the proteins on the gels were transferred onto an activated polyvinylidene difluoride (PVDF) membrane (Immobilon from Millipore, #IPFL00005) and blocked with 1X TBS-0.1% Tween-20-5% dry milk for 1 hour at room temperature. The membrane was then incubated with the corresponding primary antibody (1:5000 dilution in blocking buffer) and incubated during 1 hour at room temperature or overnight at 4°C. Blots were incubated with the following primary antibodies: anti-CHIKV nsP4 (rabbit, provided by Dr. Andres Merits), anti-CHIKV E1 (rabbit, provided by Dr. Gorben Pijlman at the University of Wageningen, Netherlands), anti-CHIKV E2 (mouse, CHIK-48; BEI Resources), anti-CHIKV capsid (rabbit, CHIK-122), and actin (mouse monoclonal, Invitrogen, #ACTN05 C4). Members were washed three times with 1X TBS-Tween-20 and the membranes were incubated with the corresponding goat anti-mouse-HRP (Invitrogen, # 31430) or anti-rabbit IgG-HRP secondary antibody (Invitrogen, # 31460) (1:10000 dilution) for 1 hour at room temperature. Members were washed extensively and developed with the Pierce SuperSignal WestPico Plus kit (ThermoScientific, # 34580). Both a chemiluminescent and a colorimetric image were taken for each membrane. Subsequently, membranes were stained with Coomassie blue and dried at room temperature to keep a trace of the protein content of each sample. Images were analyzed and quantified relative to the WT condition using ImageLab (version 6.0.1).

### Concentration of supernatants for western blot on extracellular proteins

Proteins from the culture supernatants were concentrated on Amicon columns (Amicon Ultra-0.5 Centrifugal Filter Units, cut-off of 10,000 kDa, Millipore Sigma, # UFC501024). In brief, 500 μL of sample was added on each column and spun for 15 mins at 14,000 x g at room temperature according to manufacturer’s instructions. The flow-through was discarded and each column inverted on a new tube to be reverse spun down for 15 mins at 1,000 x g at room temperature. A final volume of 20 μL purified proteins was recovered and the proteins were transferred to a new tube before adding 50 μL of lysis buffer. Samples were incubated 1 hour at 4°C and then 50 μL of 2x Laemmli with 10% β-mercaptoethanol was added to each tube. Finally, each sample was heat denatured for 10 mins at 95°C and then frozen down at -20°C until SDS-PAGE as described above.

### CHIKV full genome PCR and Sanger sequencing

For Sanger sequencing, the purified extracellular RNA was used to generate cDNA as described above. CHIKV amplicons were generated with the Phusion PCR kit (Phusion High-Fidelity DNA polymerase kit, Thermo Scientific, # F530L) and primers in **Table 3**. 5 pairs of primers were used to span the CHIKV genome (**Table 3**, CHIKV PCR primers). A 50 μL reaction was made for each sample as follow: 10 μL of 5X Phusion HF buffer, 1 μL of 10 mM dNTPs, 5 μL of 10 μM forward primer, 5 μL of 10 μM reverse primer, 0.5 μL of Phusion DNA polymerase and 4 μL of cDNA. The following PCR program was then applied: an initial denaturation at 98°C for 30 secs, then 35 cycles of a denaturation step at 98°C for 10 secs, annealing of the primers at 51°C for 30 secs and extension of the product at 72°C for 2 mins. A final extension at 72°C for 10 mins was then performed followed by a cool down at 4°C. Amplification of the fragments was then checked on a 1% agarose gel run at 120 volts for 35 mins. All positive samples were purified with the NucleoSpin Gel and PCR Clean-up kit (Macherey-Nagel, #740609.50S). Purified samples were Sanger sequenced at Genewiz (Azenta) using a specific set of primers (**Table 3**, CHIKV Sanger sequencing primers).

**Table 3:** Primers used for Sanger sequencing.

PRIMERS	Fragment	Primer name	Sequence 5’– 3’
**CHIKV PCR primers**	F1	IOLCHIK1417F	GAGGACTAGAATCAAATGG
	F1	IOLCHIK4151R	CACTGTTCTTAAAGGACTC
	F2	IOLCHIK3161F	GCATACTCACCTGAAGTAGCC
	F2	IOLCHIK4807R	AGCAGTAAGCGCAAGTGAATC
	F2.5	IOLCHIK6023F	TCATCATACCAAATTACCGACG
	F2.5	IOLCHIK8000R	GGTTTCATTACTTTGTCCCCC
	F3	IOLCHIK7203F	TTAAACTGGGCAAACCGC
	F3	IOLCHIK9807R	GCCTCTTGGTATGTGGCCGC
	F4	IOLCHIK9486F	CAACGAGCCGTATAAGTATTGG
	F4	IOLCHIK11341R	CTATTCAGGGGTTGCGTAG
**CHIKV Sanger sequencing primers (nsP4)**		IOLCHIK6444F	CACTACAGGAAGTACCAATGG
		IOLCHIK7428R	TATTTAGGACCGCCGTACAAAG

### Protein structure analysis

The CHIKV replication complex structure containing the O’nyong’nyong virus nsP4 structure (91% identity to CHIKV [21]) used in this paper is the one solved by Tan Y. B. *et al*, *Science Advances* (2022), PDB: 7Y38. The structure was visualized using PyMOL (version 2.5.2) (19).

### Alphavirus sequence alignment and consensus sequence analysis

For nsP4 alignment and WebLogo generation, 12 representative members of the alphavirus genus were selected based on Tan Y. B. *et al*, *Science Advances* (2022). Specifically, nsP4 protein sequences were first aligned using the multiple sequence alignment algorithm MUSCLE (EMBL-EMI) (19). Following are their GenBank accession numbers: CHIKV: NC_004162, ONNV: AF079456.1, SFV: NC_003215, SINV: NC_001547, RRV: GQ433354.1, MAYV: NC_003417.1, BFV: MN689034.1, VEEV: L01442.2, EEEV: EF151502.1, WEEV: MN477208.1, EILV: NC_018615.1, GETV: NC_006558.1. Using the generated multiple sequence alignment of nsP4, a consensus sequence logo was designed via WebLogo with the default color scheme. The height of each stack of letters represents the sequence conservation at the indicated position and the height of each symbol within the stack represents its relative frequency.

### Statistical analysis

Statistical analysis was performed using GraphPad Prism (version 9.3.1). A Mann-Whitney *t* test against CHIKV wild-type condition or a one-way ANOVA test between each nsP4 variants, with *p* values < 0.05 were considered significant, were performed. All experiments were done in two or more independent biological experiments and in two internal technical duplicates (details can be found in the figure legends).

## Supporting information

S1 Fig Alphaviruses nsP4 protein alignment. Twelve representative members of the Alphavirus genus (CHIKV, ONNV, SFV, SINV, RRV, MAYV, BFV, VEEV, EEEV, WEEV, EILV, GETV) were selected and nsP4 protein sequences were aligned using the multiple sequence alignment algorithm MUSCLE (EMBL-EMI). Each color represents amino acid with the same physicochemical properties (red: small hydrophobic including aromatic except Y; bleu: acidic; magenta: basic except H; green: hydroxyl, sulfhydryl, amine, glycine; grey: unusual amino acid). Symbols at the bottom of each amino acid indicates the conservation level of the position. An asterisk indicates positions which have a single, fully conserved residue. A colon indicates conservation between groups of strongly similar properties. A period indicates conservation between groups of weakly similar properties.
(TIF)

S2 Fig Nsp4 variant replication in BHK-21 cells at 37°C. BHK-21 cells from the transfections in Fig 2 were used for all the panels. (**A and B**) Intracellular proteins from transfected BHK-21 cells from a second (**A**) and third independent biological experiment (**B**) were harvested at 48 hpt. (**C**) Cells were harvested and intracellular RNA purified to quantify the amount of CHIKV RNA per μg of total RNA by RT-qPCR. Each symbol represents an independent biological experiment with solid symbols showing the nsP4 variants that reverted to the WT residue and clear symbols the variants that did not revert. A Mann-Whitney *t* test was performed against nsP4 WT, but no data reached statistical significance. Graphs show the average and SEM of four independent experiments in technical duplicates or singlet. (**D**) The active replication of each nsP4 variant was determined by quantifying the ratio of E1 CHIKV RNA (subgenomic RNA) on nsP4 CHIKV RNA (genomic RNA) by SybrGreen RT-qPCR after normalization by the 18S ribosomal RNA. A Mann-Whitney *t* test was performed against nsP4 WT but no data reached statistical significance. Graphs show the average and the SEM of four independent experiments. The genetically stable nsP4 variants names were bolded on all the panels.
(TIF)

S3 Fig CHIKV nsP4 variants are replicating in mosquito cells. Intracellular proteins from transfected C6/36 cells from a second (**A**) and third independent biological experiment (**B**) were harvested at 48 hpt. (**C**) The active replication of each nsP4 variant was determined by quantifying the ratio of E1 CHIKV RNA (subgenomic RNA) on nsP4 CHIKV RNA (genomic RNA) by SybrGreen RT-qPCR after normalization by the 18S ribosomal RNA. A Mann-Whitney *t* test was performed against nsP4 WT, but no data reached statistical significance. Graphs show the average and the SEM of four independent experiments. The genetically stable nsP4 variants names were bolded on all the panels. (**D**) Cells were harvested, and intracellular RNA purified to quantify the amount of CHIKV RNA per μg of total RNA by RT-qPCR. Each symbol represents an independent biological experiment with solid symbols showing the nsP4 variants that reverted to the WT residue and clear symbols the variants that did not revert. A Mann-Whitney *t* test was performed against nsP4 WT but no data reached statistical significance. Graphs show the average and SEM of four independent experiments in technical duplicates or singlet. (**E and F**) Quantification of E2 (**E**) and capsid (**F**) levels from immunoblots in A and B and Fig 3. One-way ANOVA was performed. *p<0.05, **p<0.01, ***p<0.001, ****p<0.0001. ns = non-significant.
(TIF)

S4 Fig CHIKV nsP4 variants in mosquito cells have an impaired release of viral structural proteins in the supernatant. Supernatants from C6/36 cells transfected with *in vitro* transcribed full-length CHIKV nsP4 variants were harvested at 48 hpt and extracellular proteins were concentrated with Amicon columns. Denatured proteins were subjected to SDS-PAGE and immunoblotted for the presence of the structural proteins capsid (top), E2 (middle) and E1 (bottom). A second (**A**) and a third (**B**) biological replicate are shown. The corresponding Coomassie blue is presented at the bottom of each Western Blot.
(TIF)

S5 Fig CHIKV intracellular and extracellular replication is sensitive to temperature. BHK-21 cells were adapted to grow at 28°C and transfected with the *in vitro* full-length CHIKV nsP4 variants for 72h. Intracellular (**A**) and extracellular (**B**) CHIKV proteins were analyzed by SDS-PAGE and immunoblotted for the protein capsid, E2, nsP4, and the house-keeping gene actin (bottom of a membrane) with molecular weights on the left side as a reference. (**C**) A kinetic of replication was done for nsP4 WT, GNN and C483Y and supernatants were taken at 24hpt, 48 hpt and 72 hpt. Infectious particles were quantified by plaque assay on Vero cells at 37°C.
(TIF)

S6 Fig Immunofluorescence negative controls and complementary nsP4 immunofluorescence images. Mock transfected C6/36 cells and transfected with WT CHIKV, the nsP4 GNN active-site variant, L368F, L368V, L383A, L383Y, and L442A stained for nsP4 (green) and DAPI (dark blue). Images are representative of two independent experiments for all panels. Scale bar: 20 μm.
(TIF)

S7 Fig Immunofluorescence images of capsid, E2, and actin for WT and nsP4 variants. Mock transfected C6/36 cells and transfected with WT CHIKV, the nsP4 GNN active-site variant, and L368F stained for DAPI (dark blue), capsid (teal), E2 (red), and actin (magenta). Images are representative of two independent experiments for all panels. Scale bar: 20 μm.
(TIF)

S8 Fig Immunofluorescence images of capsid, E2, and actin for nsP4 variants. Mock transfected C6/36 cells and transfected with L368V, L383A, L383Y, and L442A stained for DAPI (dark blue), capsid (teal), E2 (red), and actin (magenta). Images are representative of two independent experiments for all panels. Scale bar: 20 μm.
(TIF)

S9 Fig Bright-field images of BHK-21 cells at 48 hpt transfected with the *in vitro* transcribed CHIKV full-length RNA. BHK-21 cells grown at 37°C (**A**) or 28°C (**B**) are shown.
(TIF)

S1 Table Infectious virus production, subgenomic transcription and capsid protein amount relative to nsP4 WT. Table shows the mean infectious virus in PFU/mL, the mean percentage of sgRNA transcription relative to nsP4 WT (Firefly luciferase activity) and the mean capsid protein amount relative to nsP4 WT. Reversion to nsP4 is indicated by a ^**A**^, virus rescued in BHK-21 cells adapted to 28°C and titrated on Vero cells at 37°C is indicated by a ^**B**^, and virus rescued in BHK-21 cells adapted to 28°C and titrated on C6/36 cells at 28°C is indicated by a ^**C**^.
(DOCX)

S1 Data File contains all raw data available for each figure in this study. (XLSX)
