# Supplementary figures and images for: Distinct chikungunya virus polymerase palm subdomains contribute to viral protein accumulation and virion production
Source: PLoS Pathog. 2024 Oct 14;20(10):e1011972. doi: 10.1371/journal.ppat.1011972 (PMC11501042; doi:10.1371/journal.ppat.1011972)

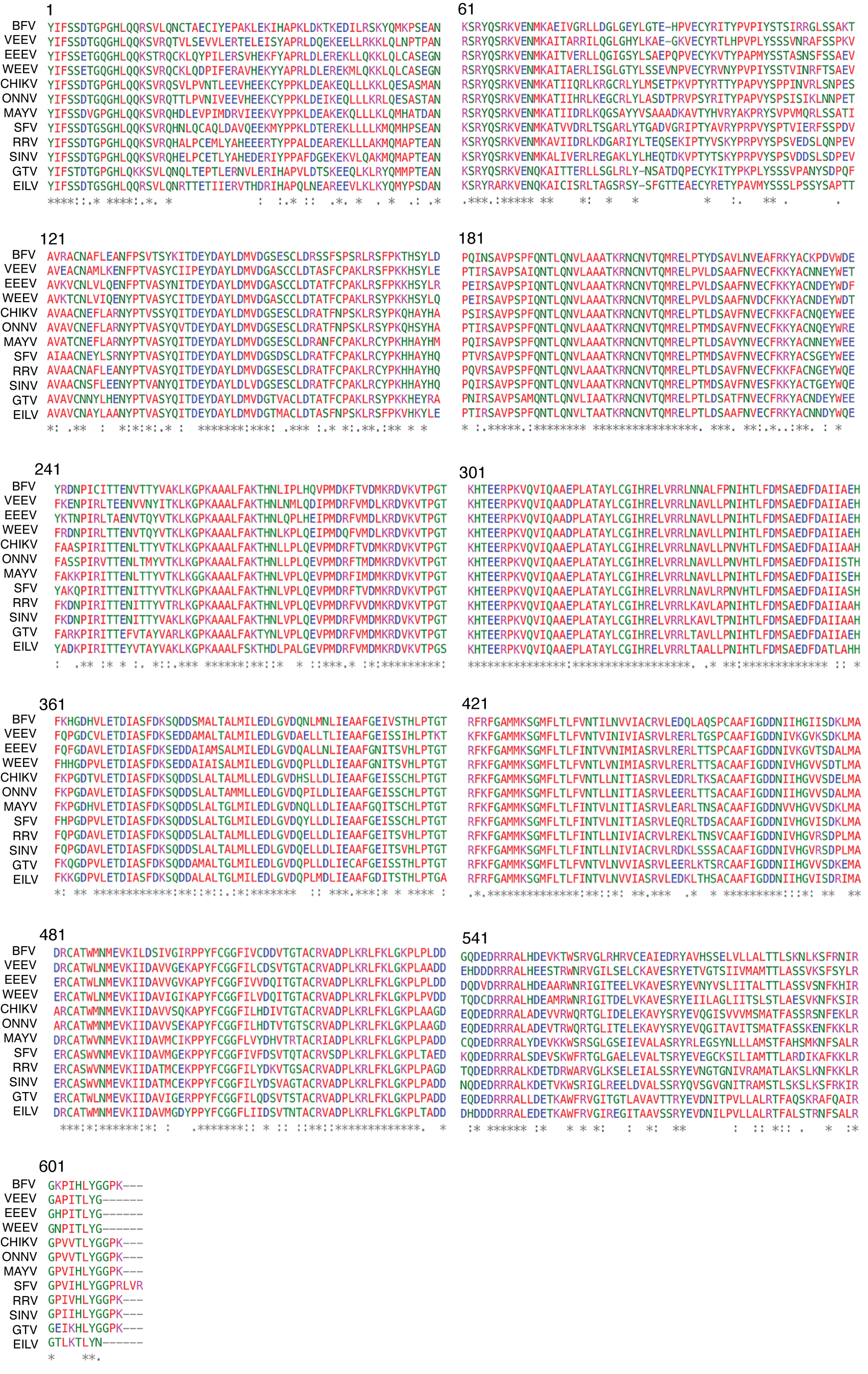

Supplement: S1 Fig — Twelve representative members of the Alphavirus genus (CHIKV, ONNV, SFV, SINV, RRV, MAYV, BFV, VEEV, EEEV, WEEV, EILV, GETV) were selected and nsP4 protein sequences were aligned using the multiple sequence alignment algorithm MUSCLE (EMBL-EMI). Each color represents amino acid with the same physicochemical properties (red: small hydrophobic including aromatic except Y; bleu: acidic; magenta: basic except H; green: hydroxyl, sulfhydryl, amine, glycine; grey: unusual amino acid). Symbols at the bottom of each amino acid indicates the conservation level of the position. An asterisk indicates positions which have a single, fully conserved residue. A colon indicates conservation between groups of strongly similar properties. A period indicates conservation between groups of weakly similar properties. (TIF) [file ppat.1011972.s001.tif]

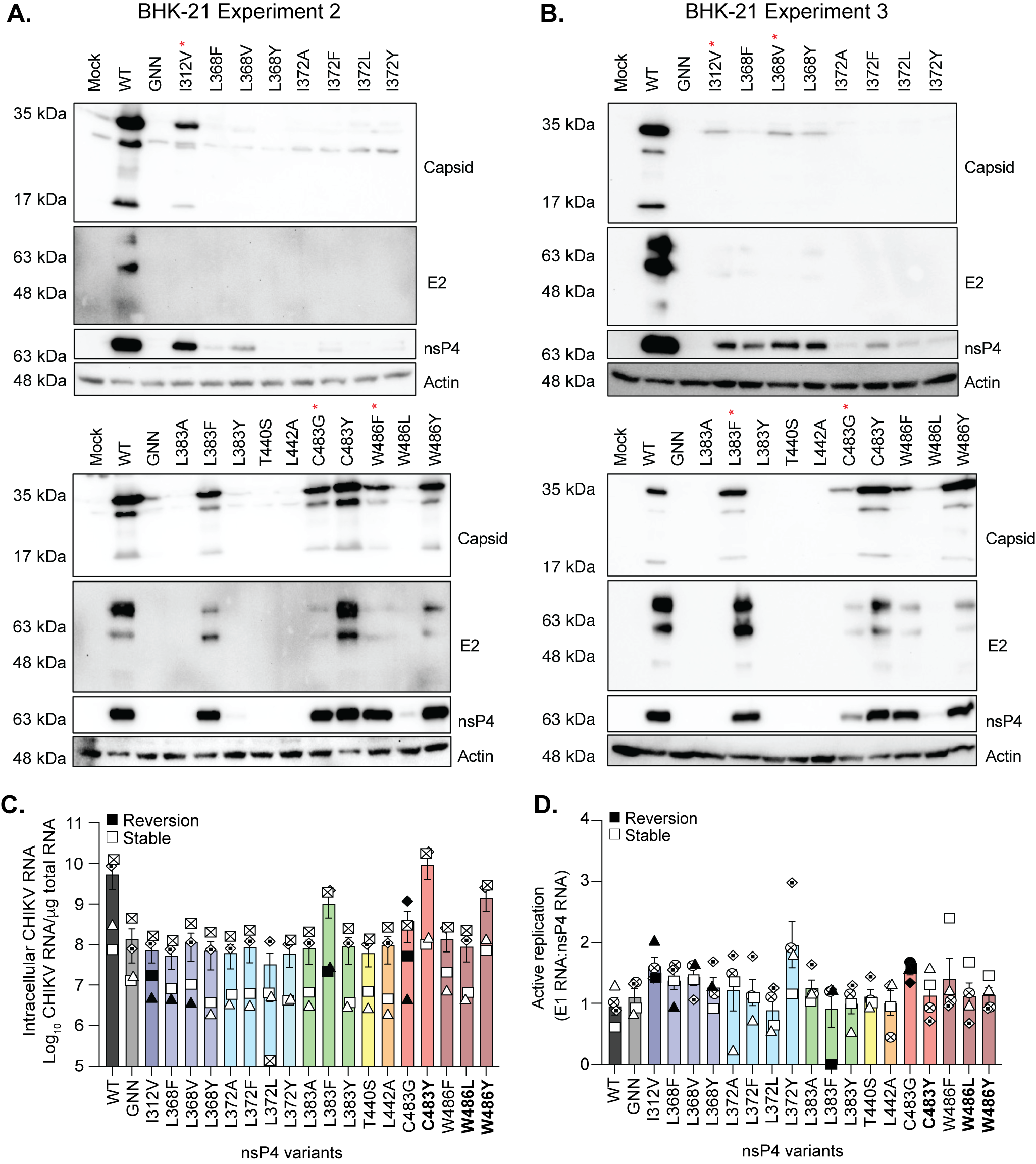

Supplement: S2 Fig — BHK-21 cells from the transfections in Fig 2 were used for all the panels. (A and B) Intracellular proteins from transfected BHK-21 cells from a second (A) and third independent biological experiment (B) were harvested at 48 hpt. (C) Cells were harvested and intracellular RNA purified to quantify the amount of CHIKV RNA per μg of total RNA by RT-qPCR. Each symbol represents an independent biological experiment with solid symbols showing the nsP4 variants that reverted to the WT residue and clear symbols the variants that did not revert. A Mann-Whitney t test was performed against nsP4 WT, but no data reached statistical significance. Graphs show the average and SEM of four independent experiments in technical duplicates or singlet. (D) The active replication of each nsP4 variant was determined by quantifying the ratio of E1 CHIKV RNA (subgenomic RNA) on nsP4 CHIKV RNA (genomic RNA) by SybrGreen RT-qPCR after normalization by the 18S ribosomal RNA. A Mann-Whitney t test was performed against nsP4 WT but no data reached statistical significance. Graphs show the average and the SEM of four independent experiments. The genetically stable nsP4 variants names were bolded on all the panels. (TIF) [file ppat.1011972.s002.tif]

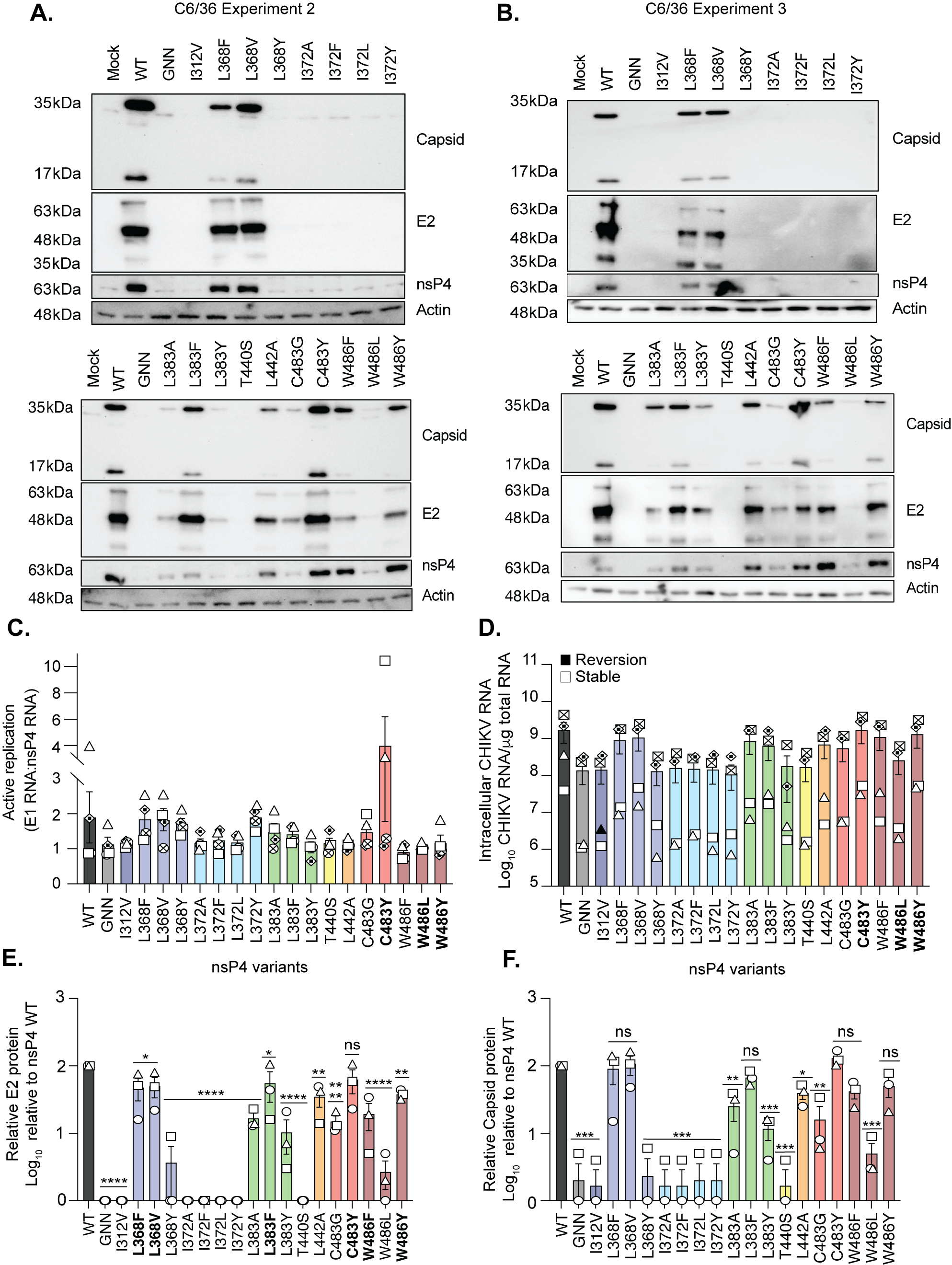

Supplement: S3 Fig — Intracellular proteins from transfected C6/36 cells from a second (A) and third independent biological experiment (B) were harvested at 48 hpt. (C) The active replication of each nsP4 variant was determined by quantifying the ratio of E1 CHIKV RNA (subgenomic RNA) on nsP4 CHIKV RNA (genomic RNA) by SybrGreen RT-qPCR after normalization by the 18S ribosomal RNA. A Mann-Whitney t test was performed against nsP4 WT, but no data reached statistical significance. Graphs show the average and the SEM of four independent experiments. The genetically stable nsP4 variants names were bolded on all the panels. (D) Cells were harvested, and intracellular RNA purified to quantify the amount of CHIKV RNA per μg of total RNA by RT-qPCR. Each symbol represents an independent biological experiment with solid symbols showing the nsP4 variants that reverted to the WT residue and clear symbols the variants that did not revert. A Mann-Whitney t test was performed against nsP4 WT but no data reached statistical significance. Graphs show the average and SEM of four independent experiments in technical duplicates or singlet. (E and F) Quantification of E2 (E) and capsid (F) levels from immunoblots in A and B and Fig 3. One-way ANOVA was performed. *p<0.05, **p<0.01, ***p<0.001, ****p<0.0001. ns = non-significant. (TIF) [file ppat.1011972.s003.tif]

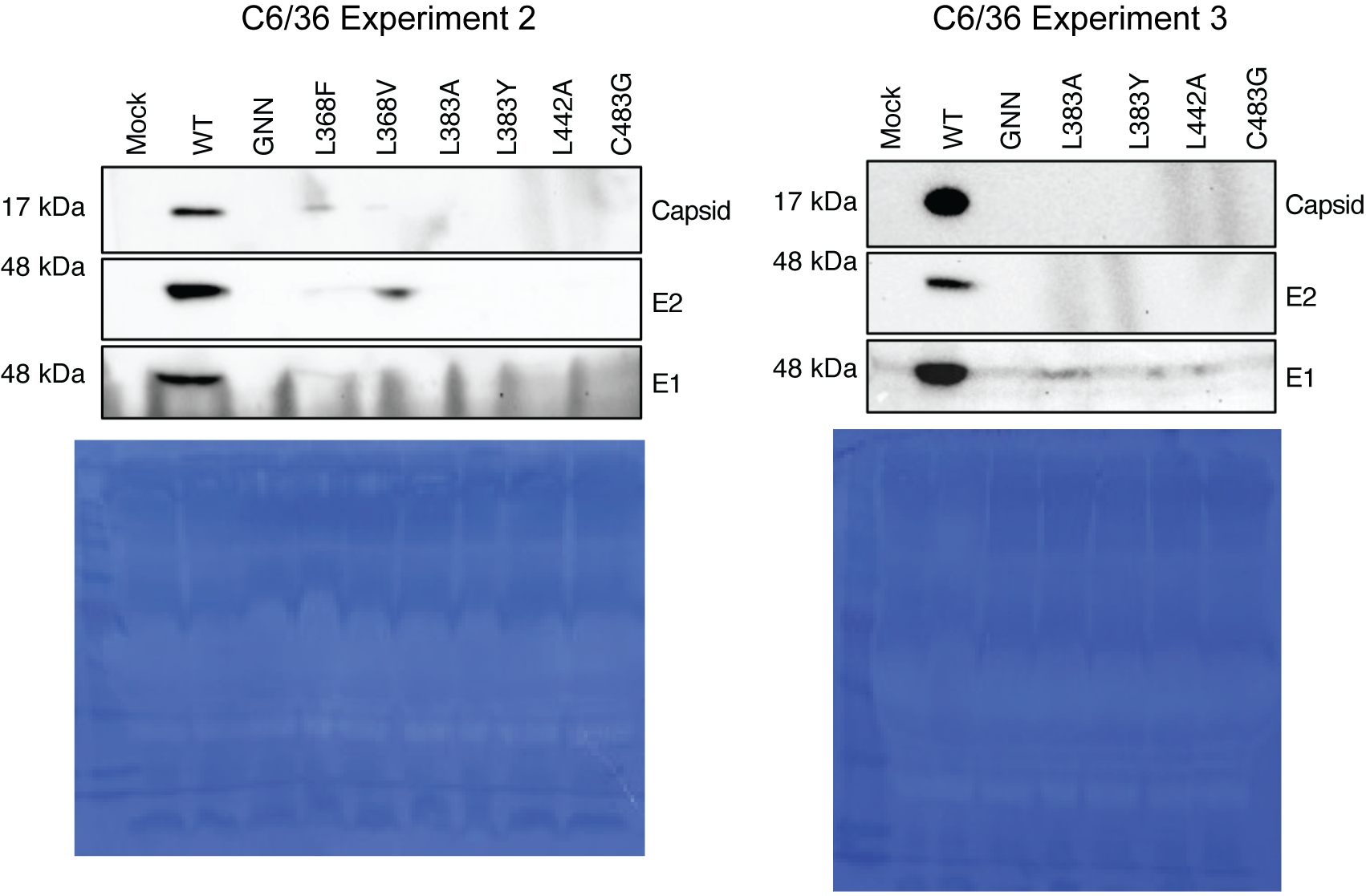

Supplement: S4 Fig — Supernatants from C6/36 cells transfected with in vitro transcribed full-length CHIKV nsP4 variants were harvested at 48 hpt and extracellular proteins were concentrated with Amicon columns. Denatured proteins were subjected to SDS-PAGE and immunoblotted for the presence of the structural proteins capsid (top), E2 (middle) and E1 (bottom). A second (A) and a third (B) biological replicate are shown. The corresponding Coomassie blue is presented at the bottom of each Western Blot. (TIF) [file ppat.1011972.s004.tif]

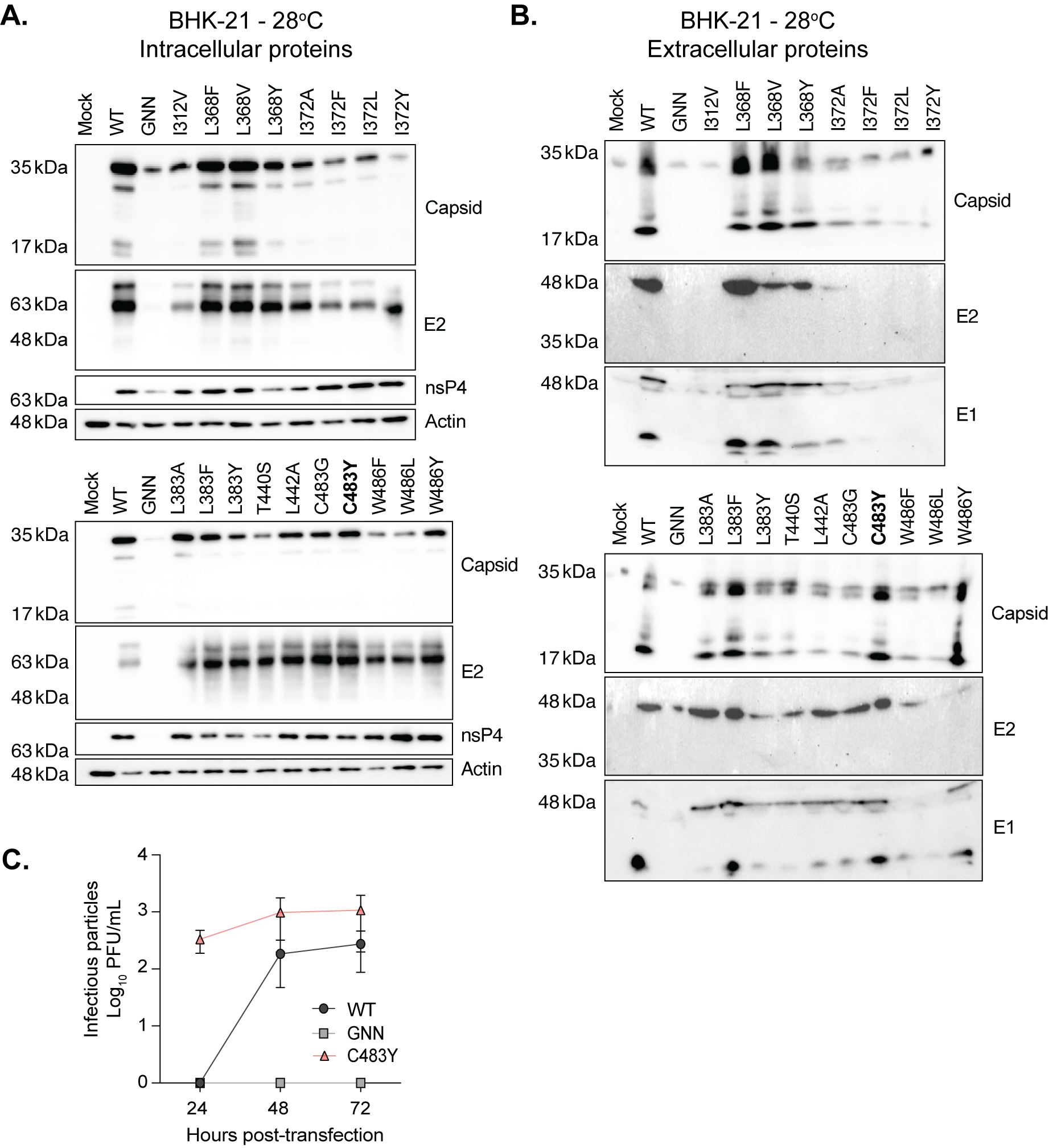

Supplement: S5 Fig — BHK-21 cells were adapted to grow at 28°C and transfected with the in vitro full-length CHIKV nsP4 variants for 72h. Intracellular (A) and extracellular (B) CHIKV proteins were analyzed by SDS-PAGE and immunoblotted for the protein capsid, E2, nsP4, and the house-keeping gene actin (bottom of a membrane) with molecular weights on the left side as a reference. (C) A kinetic of replication was done for nsP4 WT, GNN and C483Y and supernatants were taken at 24hpt, 48 hpt and 72 hpt. Infectious particles were quantified by plaque assay on Vero cells at 37°C. (TIF) [file ppat.1011972.s005.tif]

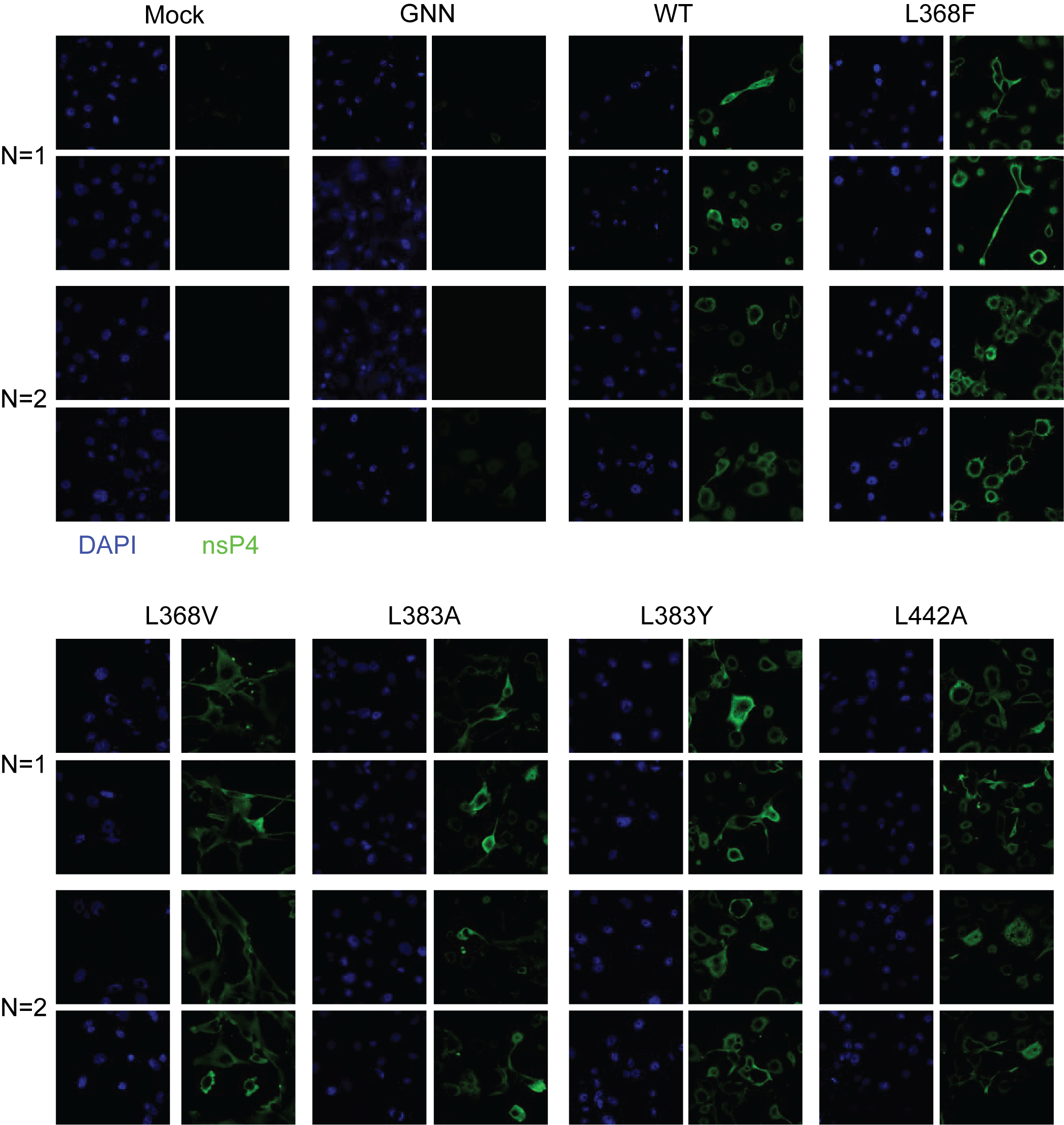

Supplement: S6 Fig — Mock transfected C6/36 cells and transfected with WT CHIKV, the nsP4 GNN active-site variant, L368F, L368V, L383A, L383Y, and L442A stained for nsP4 (green) and DAPI (dark blue). Images are representative of two independent experiments for all panels. Scale bar: 20 μm. (TIF) [file ppat.1011972.s006.tif]

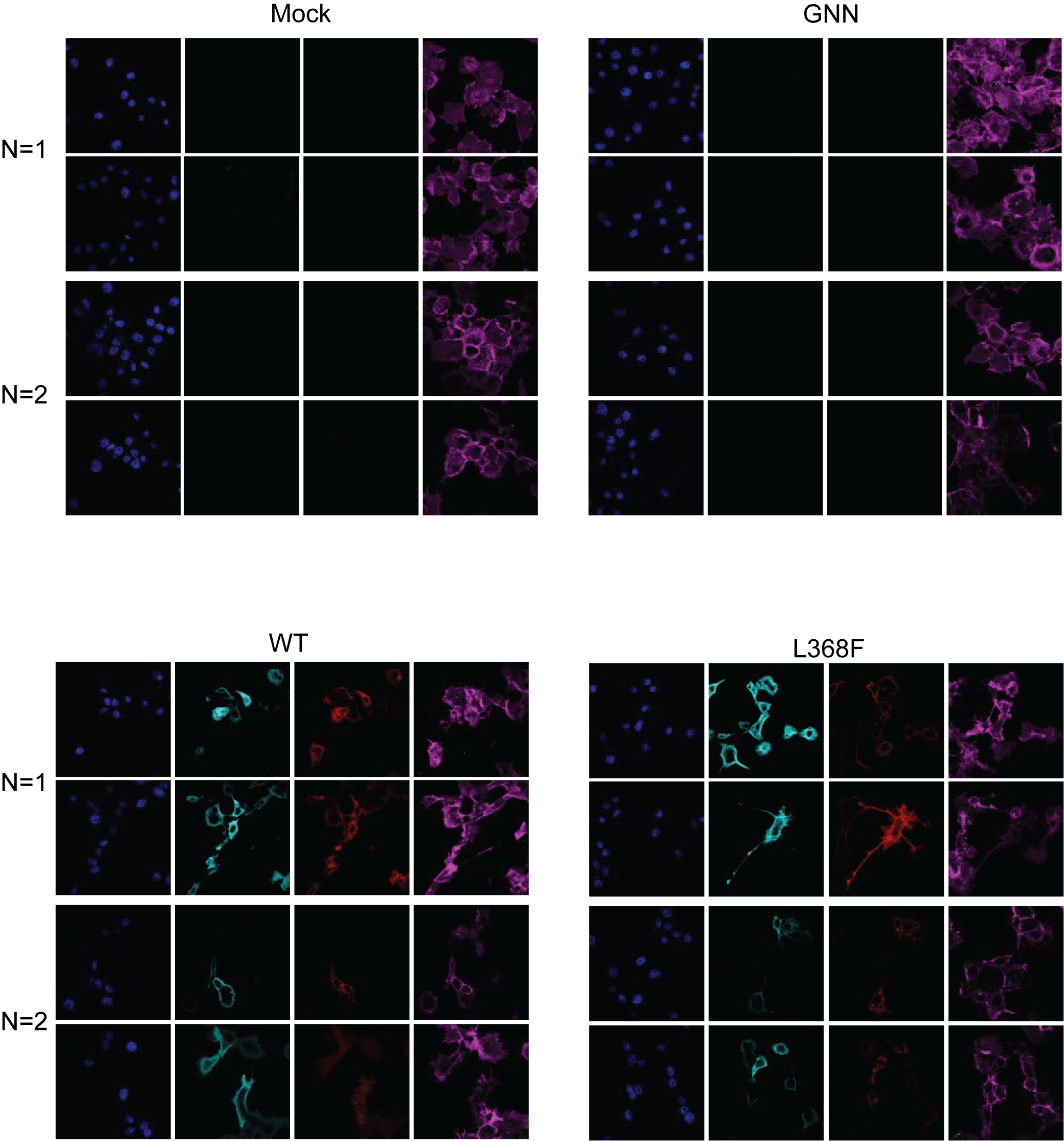

Supplement: S7 Fig — Mock transfected C6/36 cells and transfected with WT CHIKV, the nsP4 GNN active-site variant, and L368F stained for DAPI (dark blue), capsid (teal), E2 (red), and actin (magenta). Images are representative of two independent experiments for all panels. Scale bar: 20 μm. (TIF) [file ppat.1011972.s007.tif]

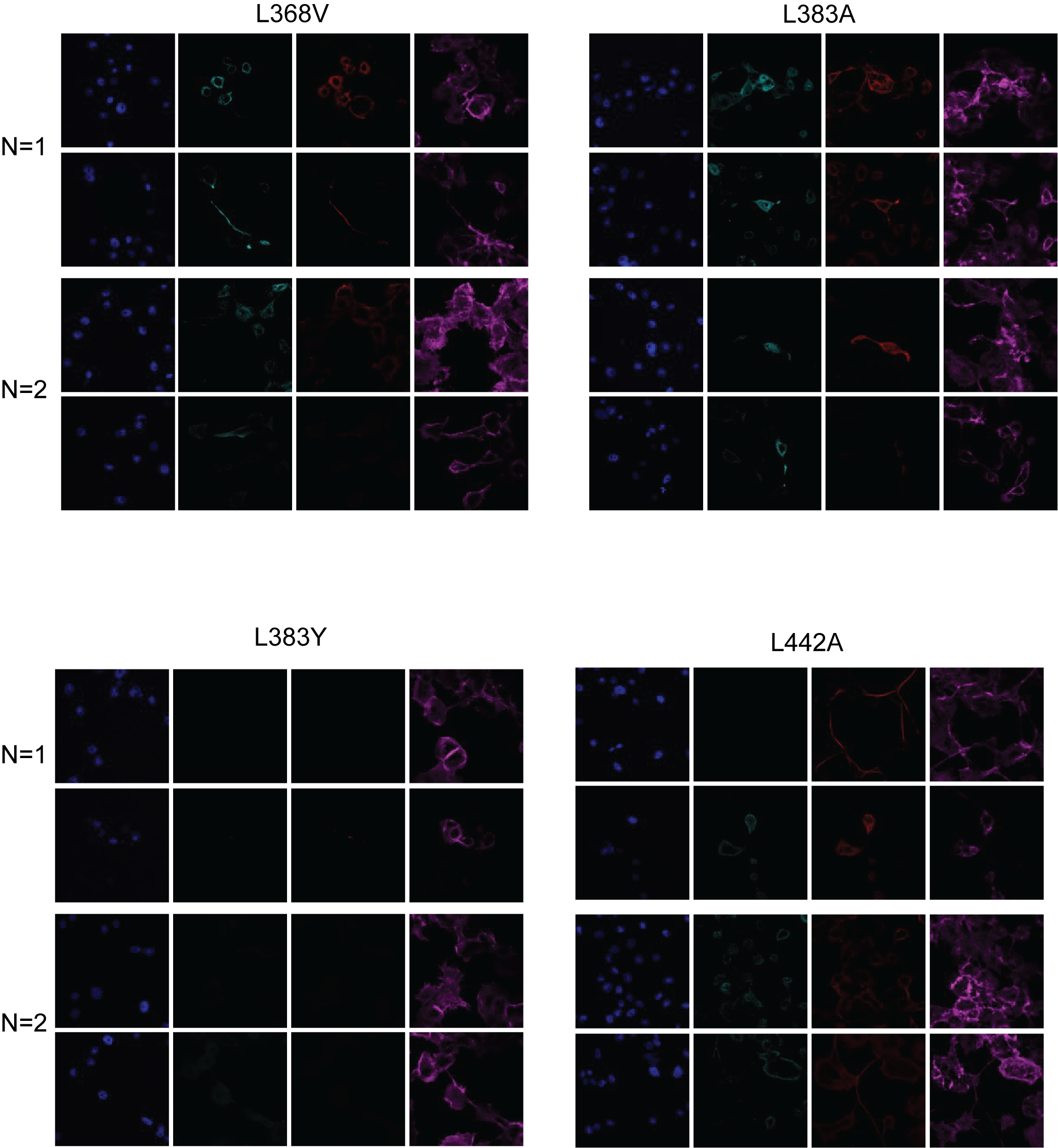

Supplement: S8 Fig — Mock transfected C6/36 cells and transfected with L368V, L383A, L383Y, and L442A stained for DAPI (dark blue), capsid (teal), E2 (red), and actin (magenta). Images are representative of two independent experiments for all panels. Scale bar: 20 μm. (TIF) [file ppat.1011972.s008.tif]

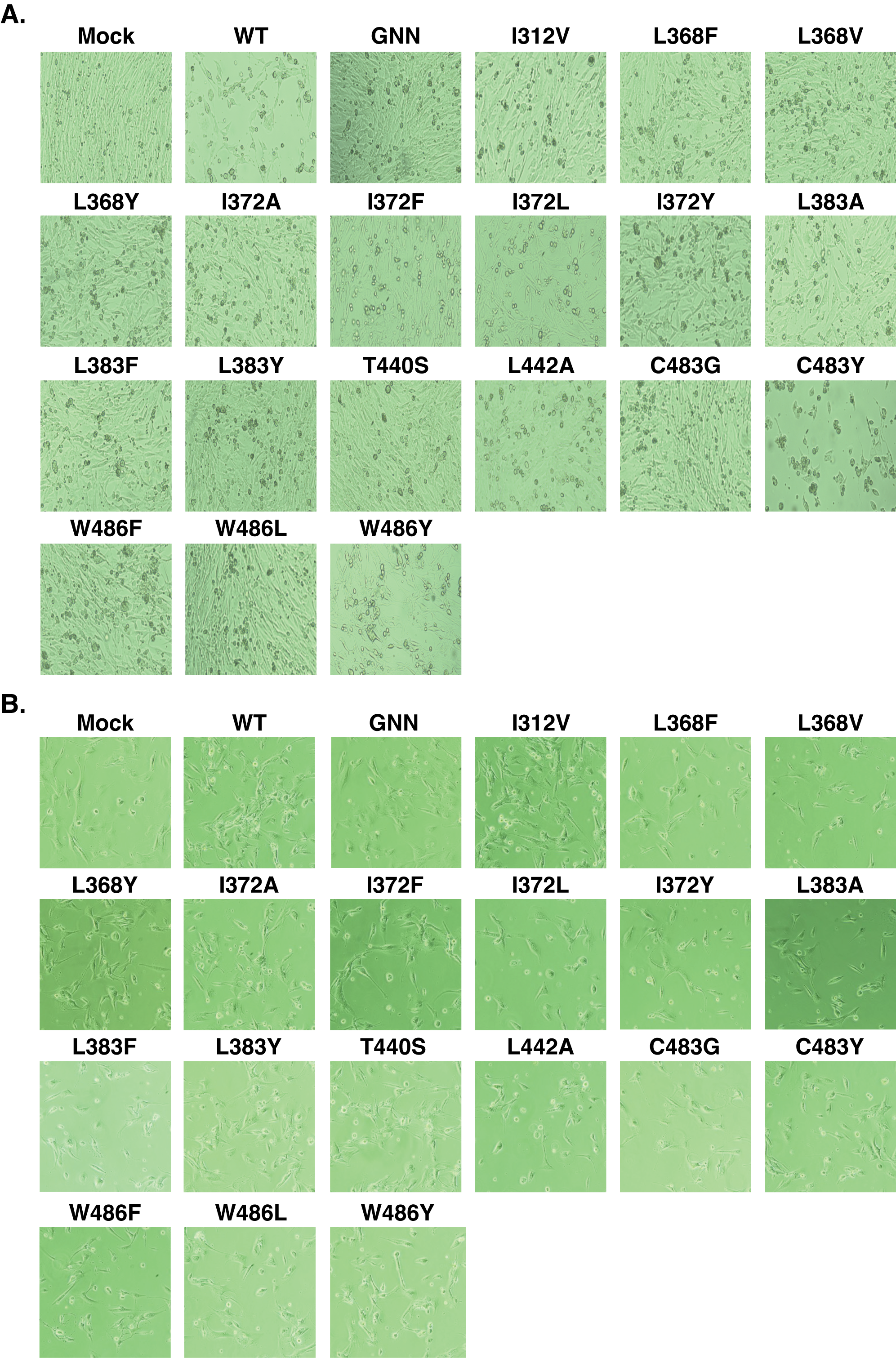

Supplement: S9 Fig — BHK-21 cells grown at 37°C (A) or 28°C (B) are shown. (TIF) [file ppat.1011972.s009.tif]
