# Supplementary material for: Distinct chikungunya virus polymerase palm subdomains contribute to viral protein accumulation and virion production
Source: PLoS Pathog. 2024 Oct 14;20(10):e1011972. doi: 10.1371/journal.ppat.1011972 (PMC11501042; doi:10.1371/journal.ppat.1011972)
Supplement: S1 Table — Table shows the mean infectious virus in PFU/mL, the mean percentage of sgRNA transcription relative to nsP4 WT (Firefly luciferase activity) and the mean capsid protein amount relative to nsP4 WT. Reversion to nsP4 is indicated by a A, virus rescued in BHK-21 cells adapted to 28°C and titrated on Vero cells at 37°C is indicated by a B, and virus rescued in BHK-21 cells adapted to 28°C and titrated on C6/36 cells at 28°C is indicated by a C. (DOCX) [file ppat.1011972.s010.docx]

­­

| **Supplemental Table 1: Summary of nsP4 variant results** | | | | | | | | | |  | | |
| --- | --- | --- | --- | --- | --- | --- | --- | --- | --- | --- | --- | --- |
| **nsP4**  **variant** | **Mean infectious**  **virus Vero cells**  **(PFU/mL)** | |  | **Mean viral particles**  **C6/36 cells**  **(TCID50/mL)** | |  | **Mean % sgRNA**  **replication of WT (subgenomic replicon)** | |  | **Capsid protein**  **(relative to WT)** | |  |
|  | **BHK-21** | **C6/36** | | **BHK-21** | **C6/36** | | **BHK-21** | **C6/36** | | **BHK-21** | **C6/36** |  |
| **WT** | 50,000 ^B^ | 250,000 | | 8,900,000^C^ | 6,700,000 | | 100 | 100 | | 1 | 1 |  |
| **GNN** | 0 | 0 | | 580,000^C^ | 17 | | 0.15 | 0.05 | | 0.01 | 0.01 |  |
| **I312V** | 1,000^A^ | 76^A^ | | 570,000^C^ | 5,700 | | 0.51 | 4.38 | | 0.15 | 0.03 |  |
| **L368F** | 17^A^ | 720 | | 7,100,000^C^ | 590,000 | | 20.21 | 249.90 | | 0 | 0.86 |  |
| **L368V** | 120^A^ | 51 | | 7,900,000^C^ | 69,000 | | 8.90 | 245.50 | | 0.08 | 0.92 |  |
| **L368Y** | 0 | 0 | | 490,000^C^ | 530 | | 0.10 | 127.62 | | 0.01 | 0.03 |  |
| **I372A** | 0 | 0 | | 5,300,000^C^ | 45 | | 0.18 | 29.20 | | 0 | 0.01 |  |
| **I372F** | 0 | 0 | | 550,000^C^ | 27 | | 4.73 | 49.65 | | 0 | 0.01 |  |
| **I372L** | 0 | 0 | | 4,800,000^C^ | 11 | | 0.04 | 0.89 | | 0 | 0.01 |  |
| **I372Y** | 0 | 0 | | 61,000^C^ | 0 | | 0.15 | 17.00 | | 0 | 0.01 |  |
| **L383A** | 0 | 0 | | 7,300,000^C^ | 750,000 | | 2.45 | 103.46 | | 0.01 | 0.23 |  |
| **L383F** | 6,200^A^ | 2,300 | | 7,700,000^C^ | 4,600,000 | | 13.48 | 38.74 | | 0.89 | 0.99 |  |
| **L383Y** | 0 | 0 | | 690,000^C^ | 5,300 | | 4.31 | 99.06 | | 0.01 | 0.10 |  |
| **T440S** | 34^A^ | 0 | | 6,400,000^C^ | 630 | | 0.16 | 24.20 | | 0 | 0.01 |  |
| **L442A** | 0 | 0 | | 6,800,000^C^ | 770,000 | | 0.36 | 88.53 | | 0.01 | 0.01 |  |
| **C483G** | 14,000^A^ | 12 | | 7,100,000^C^ | 63,000 | | 4.84 | 116.39 | | 0.39 | 0.28 |  |
| **C483Y** | 26,000^B^ | 400,000 | | 9,400,000^C^ | 6,000,000 | | 39.23 | 372.22 | | 1.52 | 1.67 |  |
| **W486F** | 1,700^A^ | 4,500 | | 6,900,000^C^ | 86,000 | | 15.52 | 42.35 | | 0.56 | 0.50 |  |
| **W486L** | 59 | 0 | | 7,300,000^C^ | 6,900 | | 7.30 | 34.92 | | 0.07 | 0.09 |  |
| **W486Y** | 10,000 | 4,900 | | 8,800,000^C^ | 5,200,000 | | 25.40 | 113.93 | | 2.04 | 1.89 |  |

A: Reversion to nsP4 WT

B: Rescued in BHK-21 cells at 28^o^C on Vero cells

C: Rescued in BHK-21 cells at 28^o^C on C6/36 cells
